# Reactivity Assessment of Diverse Aluminosilicate Wastes in Metakaolin-Based Alkali-Activated Binders

**DOI:** 10.3390/ma19183900

**Published:** 2026-09-14

**Authors:** Victorien Bienvenu Abanda Well, Mattia Giovini, Francesco Genua, Isabella Lancellotti, Cristina Leonelli

**Affiliations:** Department of Engineering “Enzo Ferrari”, University of Modena and Reggio Emilia, 41125 Modena, Italy

**Keywords:** alkali activation, industrial waste, compressive strength, reactivity degree, chemical stability

## Abstract

**Highlights:**

- Comparative cementing activity of five industrial wastes was assessed in alkali-activated binders.
- Float-glass polishing sludge showed the best performance (~25 MPa at 10 wt% addition), ahead of both bottom and fly ash.
- Compressive strength was correlated with crystallinity and Si/Al molar ratio.
- XRD, FT-IR and SEM supported the classification of wastes according to cementing activity.
- The findings support sustainable waste valorization in alkali-activated materials.

**Abstract:**

The development of carbon-neutral construction materials has stimulated interest in alkali-activated systems for the valorization of industrial non-hazardous waste. This study proposes an original comparative approach to assess the cementing reactivity of several wastes, including black and white fly ash, bottom ash, fine glass dust, and float-glass polishing sludge, through their use as partial replacements for metakaolin (MK). Formulations containing 5–50 wt% of fine waste powders (<45 μm) were prepared and mechanically compared with a reference MK-based geopolymer. Formulations containing waste additions to the reference geopolymeric paste were also evaluated to investigate their role as aggregates/fillers. Mechanical testing identified float-glass polishing sludge as the most reactive precursor, achieving a compressive strength of 25 MPa at 10 w% addition, compared with 16 MPa for the reference material. Bottom ash and black and white fly ash reached approximately 19–21 MPa at 5–10% replacement or addition. Conversely, bottom ash at substitution levels above 5% reduced mechanical performance owing to its high crystallinity and unfavorable Si/Al molar ratio. Microstructural characterization by XRD, FT-IR, density measurements, and SEM was correlated with the observed cementing activity. These results provide a basis for performance-based design criteria aimed at the sustainable valorization of locally available industrial by-products in alkali-activated materials.

## 1. Introduction

The development of sustainable urban infrastructure has made the search for alternatives to ordinary Portland cement (OPC) a global priority [1]. While geopolymers have emerged as a promising cement-free alternative with a significantly lower carbon footprint, their viability for structural applications is primarily determined by their compressive strength [2]. Geopolymers, often identified as alkali-activated inorganic binders, are produced by the chemical activation of aluminosilicate precursors in highly alkaline solutions. The resulting three-dimensional aluminosilicate framework is characterized by high mechanical strength, low permeability, and excellent durability. Owing to their intrinsic thermal stability and superior resistance to acidic and chemically aggressive environments, geopolymer binders have emerged as promising alternatives to ordinary Portland cement (OPC) for demanding applications, including fire-resistant materials for road and railway tunnels, refractory components, sewer pipes, and wastewater infrastructures. In these environments, the performance of OPC may be significantly affected by thermal degradation or acid attack, whereas geopolymer matrices generally retain a greater fraction of their structural integrity and mechanical properties [2,3,4]. Achieving high-strength geopolymeric binders requires the optimization of several complex variables, ranging from the mineralogical composition of the precursors to the chemical parameters of the activating solutions [3,4].

A critical factor in controlling strength development is the SiO_2_/Al_2_O_3_ molar ratio within the geopolymeric matrix [5]. Research indicates that compressive strength typically increases with this ratio until it reaches an optimal peak, often around 3.5, before declining due to the saturation of the bulk solution, which can hinder precursor dissolution [1]. Furthermore, the source of these oxides is vital; studies have shown that soluble silica supplied externally via sodium silicate (Na_2_SiO_3_) more effectively promotes a densely packed, high-strength network than relying solely on the silica present in raw materials like metakaolin or bottom ash [6].

The chemical activation process also plays a decisive role in the final strength results. Increasing the NaOH molarity generally accelerates geopolymerization and enhances the extraction of reactive alumina and silica species, leading to higher compressive strength [7]. However, exceeding an ideal limit (often around 14–16 M) can lead to a decrease in strength due to the premature precipitation of gel phases [4]. Other strong bases, such as potassium hydroxide (KOH) and lithium hydroxide (LiOH), play distinct roles in geopolymer structure and development due to the size and charge density of their respective alkali metal cations (K^+^ and Li^+^) [8]. Although workability increases with the ionic radius of the activating cation (Li < Na < K), compressive strength remains the key parameter for assessing the overall performance of the binders [8]. The highest strength values depend on the precursor composition, with NaOH-performing systems showing superior strength in GGBS-rich mixtures, KOH being more effective in GGBS-only binders, and LiOH achieving the highest strength in FA-only formulations. Considering its consistently high mechanical performance across different binder chemistries, together with its substantially lower cost and lower environmental impact compared with LiOH and KOH, NaOH represents the most advantageous activator for practical applications.

The experimental evaluation of reactivity in alkaline environments remains the most reliable method, given the large number of variables affecting the performance of the final material. For Supplementary Cementitious Materials (SCMs), a RILEM Technical Committee proposed assessing reactivity through the 28-day relative compressive strength of standard mortar bars in which 30 wt% of the cement is replaced by the SCM under investigation [9].

This preliminary study focuses on the evaluation of the compressive strength of geopolymer formulations incorporating five different inorganic industrial waste streams, including two fly ashes and one bottom ash from a biomass-based energy plant, and glass-derived residues from a glass container recycling process. As an initial screening approach, the finest fraction of each waste material (particle size < 45 μm) was selected by simple sieving of the as-received industrial by-products. These fractions were then used either as partial replacements for or additions to metakaolin (MK) in a reference formulation previously optimized and validated in earlier studies [10,11,12,13]. We chose a commercial, ready-to-use metakaolin for easier reproducibility of our experiments.

Geopolymer samples were prepared and cured at room temperature in order to minimize energy consumption and maintain a low-energy processing route. By investigating the interactions between waste-derived particles, metakaolin, and the alkaline activating solution, this study aims to identify mix designs capable of achieving improved mechanical performance while providing a simple, rapid, and cost-effective methodology for assessing the reactivity of aluminosilicate waste sources.

In addition, the mechanisms governing strength development were investigated through an integrated experimental approach combining compressive strength measurements, FT-IR spectroscopy, SEM-EDS analysis, and chemical stability tests, enabling the correlation of mechanical performance with the microstructural and chemical features of the resulting alkali-activated binders [3].

## 2. Materials and Methods

### 2.1. Raw Materials and Wastes

In the present study, the mix design was based on a metakaolin (MK)-based geopolymer formulation previously developed and optimized in our earlier investigations [10,11,12,13]. The metakaolin employed was a commercial product typically used for low carbon cement, mortars and concrete, as well as lime mortars and plasters (ARGICAL™ M–1000, Imerys, Paris, France) with the following chemical composition (wt%): SiO_2_ = 55.0, Al_2_O_3_ = 40.0, Fe_2_O_3_ = 1.4, TiO_2_ = 1.5, Na_2_O + K_2_O = 0.8, CaO + MgO = 0.3, and loss on ignition (LOI) = 1.0, d_50_ = 6 μm; B.E.T. = 20 m^2^/g (data from the manufacturer). This metakaolin is widely used as a reference precursor in geopolymer research due to its well-defined chemical composition, high purity, and excellent mineralogical and microstructural stability [14]. From a mineralogical perspective, the material is mainly composed of a highly reactive amorphous aluminosilicate phase, which constitutes the metakaolin structural network, together with minor amounts of crystalline impurities, including quartz, anatase, and traces of muscovite [15]. The predominance of the amorphous aluminosilicate phase provides high reactivity under alkaline activation conditions, making this material particularly suitable as a benchmark precursor for the evaluation of alternative aluminosilicate sources and industrial waste-derived feedstocks [16].

The alkaline activating solution was prepared by combining a commercial sodium silicate solution (Ingessil, Verona, Italy) with an 8 M sodium hydroxide (NaOH) solution prepared from laboratory-grade NaOH granules (96 wt%, Sigma-Aldrich, Milano, Italy). The sodium silicate solution was characterized by a SiO_2_/Na_2_O molar ratio of 3.0, a pH of 11.7, and a density of 1.368 g cm^−3^ at 20 °C. The 8 M NaOH solution was prepared by gradually dissolving the NaOH granules in distilled water under continuous stirring. The alkali was added slowly to control the heat released by the highly exothermic dissolution process and to minimize water evaporation. Upon completion of the dissolution step, the container was sealed and allowed to cool to room temperature. After cooling, the sodium silicate solution was added at a sodium silicate-to-sodium hydroxide mass ratio of 1.05, corresponding approximately to a 50:50 volumetric ratio. The resulting activating solution was homogenized in a rotary mixer for 24 h prior to its use in the preparation of the geopolymer formulations.

To assess the reactivity of the investigated waste-derived powders, a reference geopolymer formulation containing a conventional inert aggregate was also prepared. Standardized sand was added to the fresh geopolymer paste, and the resulting mechanical properties were compared with those obtained using the fine waste fractions. The standardized sand employed in this study was a commercial product supplied by SNL (Paris, France).

Five different non-hazardous inorganic waste streams characterized by varying amounts of Si- and Al-bearing phases were selected for this study. The investigated non-hazardous industrial by-products included float-glass polishing sludge, fine glass dust, black and white fly ash, and bottom ash. In addition to differences in chemical composition, the wastes exhibited significant variability in their degree of crystallinity and in the content of secondary oxide components, particularly CaO, which is known to strongly influence alkali activation processes. All waste materials were classified according to the European List of Waste (LoW) and identified by their corresponding European Waste Catalogue (EWC/EER) codes [17]:

• Float-glass polishing sludge (EER 10 11 12): Waste generated during the manufacture of float glass. Specifically, this material originates from the filter-press treatment of polishing wastewater and is classified as non-hazardous.
• Fine glass dust (EER 19 12 05): Glass-rich residue derived from the mechanical treatment of separately collected municipal glass waste, including sorting, crushing, compaction, and related processing operations.
• Black and white fly ash (EER 10 01 03): Fly ash generated from the combustion of biomass fuels, including peat and untreated wood, for energy production.
• Bottom ash (EER 19 01 12). Non-hazardous bottom ash and slag originating from waste incineration processes.

The selected waste streams were intentionally chosen to represent a broad range of chemical compositions, mineralogical assemblages, and amorphous phase contents, enabling a systematic assessment of their potential reactivity in alkali-activated systems.

The first stage of alkali-activated binder preparation involved the pre-treatment of the as-received industrial wastes. All waste materials were first homogenized and oven-dried at 110 °C for 24 h to remove residual moisture. Subsequently, the dried materials were ground (except for the white fly ash, which was already sufficiently fine) and sieved to a particle size below 45 μm, following the recommendations of the ASTM C430-25 standard [18]. The selection of the finest particle fraction was intended to maximize the potential reactivity of the waste materials and to enable a meaningful comparison among different waste streams under identical alkaline activation conditions. Furthermore, the use of a simple sieving procedure allowed the investigation of waste reactivity without the need for additional energy-intensive treatments such as grinding or milling, thereby preserving the low-cost and sustainable nature of the proposed approach.

The resulting fine powders obtained from the five waste materials were subsequently characterized according to the procedures described in Section 2.3.

### 2.2. Alkali Activation and Sample Preparation

A reference geopolymer formulation (GP0) was prepared according to a mix design previously optimized and validated in earlier studies [10,11,12,13].

In the present work, the effect of replacing metakaolin (MK) with different industrial waste materials was systematically investigated. For each waste stream, the maximum replacement level was determined on the basis of the workability of the fresh paste while maintaining the same liquid-to-solid ratio adopted for the reference formulation. According to this criterion, metakaolin substitution levels of 5, 10, 25, 50, and 100 wt% (with respect to the dry MK mass) were selected for experimental evaluation (Table 1). For identification purposes, the alkali-activated materials were labeled according to the code S-WNO, where W denotes the waste source and NO indicates the substitution level (wt% replacement of metakaolin).

In addition to the replacement approach, a second series of formulations was designed to investigate the behavior of the waste materials when acting as fillers or aggregate-like components. In this case, each waste was added directly to the fresh GP0 paste, and the maximum addition level was again established according to workability requirements. The resulting addition levels were 5, 10, and 15 wt% relative to the mass of the fresh GP0 paste (Table 2). The alkali-activated materials produced in this study were labeled as R3-WNO, where W indicates the type of waste used and NO represents the addition level.

To provide a benchmark for the effect of an inert particulate addition, standardized sand was incorporated into the GP0 formulation at the same addition levels. This comparison enabled the distinction between the contribution arising from the potential chemical reactivity of the waste materials and the effect associated with the incorporation of a conventional inert aggregate on the mechanical performance of the resulting alkali-activated binders.

The resulting mixtures were singularly homogenized in a planetary mixer (AUCMA 1400 W, AUCMA Co., Ltd., Qingdao, China) for 3 min. The fresh paste was then cast into cylindrical plastic molds, sealed for 24 h to prevent moisture loss, then demolded and cured at room temperature for 28 and 51 days. The testing age of 51 days was selected due to laboratory scheduling constraints associated with the availability of the mechanical testing equipment. Nevertheless, as the study focuses on a comparative assessment, all specimens were tested at the same curing age. Therefore, any influence of curing time was consistent across all samples and did not affect the validity of the comparison among the investigated formulations.

Three specimens were prepared for each formulation in order to evaluate the variability and reproducibility of the experimental results.

### 2.3. Sample Characterization

#### 2.3.1. Industrial Waste Characterization

The five waste materials were characterized in terms of chemical composition by wavelength-dispersive X-ray fluorescence (WD-XRF) using a Zetium XRF spectrometer (Malvern Panalytical, Malvern, Worcestershire, UK) equipped with the “Omnian” software package. This software provides a standardless, semi-quantitative elemental analysis and is particularly suitable for the analysis of pressed powder specimens. The method delivers reliable results, with a typical relative accuracy ranging from ±5% to ±10% for most matrices.

Regarding mineralogical characterization, the crystalline phases present in the samples were investigated by X-ray diffraction (XRD) using Cu Kα radiation (λ = 1.5406 Å) generated at 40 kV and 40 mA. Diffraction patterns were collected over a 2θ range of 5–90° with a step size of 0.013° under continuous scanning conditions. Measurements were performed at ambient temperature using a fixed divergence slit of 0.25° and a receiving slit of 0.10 mm. No sample spinning or incident-beam monochromator was employed during data acquisition.

#### 2.3.2. Alkali-Activated Binder Characterization

All consolidated products were initially evaluated for their chemical stability prior to further characterization. Chemical resistance was assessed through a dissolution test previously reported in the literature [19]. A specimen of the consolidated geopolymeric binder was immersed in distilled water at a solid-to-liquid ratio of 1:100 for 24 h. After immersion, the specimen was removed from the solution and mechanically tested by applying pressure with pliers to evaluate its structural integrity. Formulations that retained their mechanical rigidity and showed no evidence of dissolution or degradation were considered adequately consolidated and selected for subsequent characterization.

The chemical stability of the alkali-activated materials was further assessed by measuring the pH and ionic conductivity of the eluate obtained after immersing a geopolymer specimen in distilled water at a solid-to-liquid ratio of 1:10 for 24 h (according to EN12457-2) [20]). The pH of the leachate was measured using an electrode particularly effective in titrations with strong acids and bases (Hamilton Liq-Glass SL pH electrode, Hamilton Company, Bonaduz, Switzerland), while ionic conductivity was determined using a COND 6/TDS 6 conductivity meter (Eutech Instruments Pte Ltd., Singapore). These parameters provide indirect information on the concentration of hydroxyl ions (pH) and dissolved ionic species (ionic conductivity) released into the solution as a result of unreacted or weakly bound compounds remaining in the alkali-activated matrix [12,21]. Consequently, pH and conductivity measurements can be considered indirect indicators of the extent of geopolymerization and polycondensation reactions. Based on preliminary tests involving repeated preparation and characterization of the waste-containing geopolymer binders, the reproducibility error, expressed as the maximum percentage deviation among replicate measurements, was found to range from ±0.98 to ±2.3% for pH and from ±3.8 to ±13.4% for the ionic conductivity of the eluates.

Similarly to the waste materials, all alkali-activated binders were characterized by X-ray diffraction (XRD) to identify the crystalline phases present in the consolidated products. The analytical procedure described in Section 2.3.1 was therefore repeated for all geopolymer specimens.

The morphological analysis of the alkali-activated binders after 51 days of curing was conducted by scanning electron microscopy (SEM) using an ESEM Quanta 200 microscope (FEI, Hillsboro, OR, USA) using a backscattered electron detector. Elemental analyses were performed using an Oxford Instruments EDS system controlled by INCA-350 software (Oxford Instruments NanoAnalysis, High Wycombe, UK) coupled to the SEM. Freshly fractured surfaces were sputter-coated with a thin gold layer before imaging.

All consolidated binders that successfully passed the integrity test were subsequently evaluated for their compressive mechanical performance using three replicate specimens per formulation.

Fourier transform infrared (FT-IR) spectroscopy was employed to evaluate variations in the degree of geopolymerization between the reference formulation (GP0) and the formulations containing waste materials as either additives or partial metakaolin substitutes. Spectra were collected using a Prestige-21 Shimadzu spectrophotometer (Shimadzu Italia S.r.l., Milan, Italy) equipped with a Golden Gate^®^ Diamond ATR accessory (Specac, Orpington, UK) over a wavenumber range of 4000–500 cm^−1^. The obtained spectra were subsequently analyzed using Spectragryph 1.2 software to identify structural modifications associated with the geopolymerization process and to correlate them with the incorporation of the investigated waste materials.

Compressive strength tests were performed on cylindrical specimens measuring 37 mm in diameter and about 60 mm in height (accordingly to ASTM C39 [22]) using an Instron 5567 Universal Testing Machine (Norwood, MA, USA). The load was applied incrementally at a crosshead rate corresponding to 2400 N min^−1^ up to a maximum load of 37.7 kN. Prior to testing, all specimens were dry-ground to obtain flat and parallel (within a tolerance of 0.050 mm) loading surfaces, thereby minimizing stress concentrations and ensuring a uniform load distribution during compression. When the specimen length-to-diameter ratio was less than 2.0, correction to the compressive strength value was performed by multiplying the result of the test by the appropriate correction factor accordingly to ASTM C39 [22]. Three specimens were tested for each formulation.

## 3. Results

### 3.1. Characterization of the Five Wastes

Chemical compositions of the five wastes are reported in Table 3 as far as the major oxides are concerned. Trace elements, in ppm concentrations, are reported in Table S1 of the Supplementary Materials. It should be remembered that the as-received wastes were simply dried, ground (in some extends) and sieved below 45 μm; thus, this fine fraction is not entirely representative of the chemical composition of each waste stream.

It can be observed that the SiO_2_ content is higher for MO waste (first row of Table 3) and for fine glass dust (PA) because both these wastes come directly from the glass production. The lowest silica content is recorded for FAB. MO chemical composition is in line with that of float glass as reported in the literature [23,24].

A second element varies uniformly from the first row to the last one: CaO. In this case, the content of calcium oxide increases from MO to FAB, with FAN being closer to Class F ash and FAB closer to Class C ash.

From this observation we can expect that the alkali activation will lead to Na_2_O-Al_2_O_3_-SiO_2_-H_2_O (N-A-S-H) geopolymer phase for the first wastes in Table 3, while for the wastes in the three bottom lines, the presence of high CaO contents might lead to the formation of CaO-SiO_2_-H_2_O (C-S-H) gel phase mixed with N-A-S-H. Such a mixed gel is very efficient in consolidating the alkali-activated structure of the final consolidated materials [25,26,27,28,29].

The aluminum content does not exhibit the same trend as observed for Si and Ca. High Al concentrations were found in both FAN and BA. While the elevated Al content in BA is expected, its high concentration in FAN is particularly unusual, although it is consistent with the results reported by Li et al. in a very recent work [30]. As commented above, the investigated fraction is not representative of the whole sample, but only of the finest portion.

Alkali elements are mainly present as Na in the glass wastes (MO and PA) [31], owing to its use in glass formulations to reduce the melting temperature. In contrast, K is the predominant alkali element in the biomass-derived wastes (FAN and FAB), as reported by Li et al., who found a K content of 8.43% [30]. Only in the second case do we expect to find easily leachable cations, while we do expect lower release as a consequence of the higher chemical stability of glass-derived waste streams. This is confirmed by the following XRD, where potassium contained in glass waste is inside the glass network, while for biomass ash it is present as salts (sulphates and chlorides).

The amount of Fe is particularly high in incinerator bottom ash in comparison to data from the literature corresponding to 4–6%, while for this waste the presence of P_2_O_5_ is less encountered [27,32].

Phosphates are also present in the two fly ashes due to their presence in vegetable species [33,34].

Sulfur is particularly high in FAB, presumably derived from original biomass, as are phosphates and potassium [34].

Cerium was not detected in significant amounts in MO (approx. 400 ppm); this suggests that SiC-based polishing powders [35] were used instead of CeO_2_-based polishing agents [36] during the industrial finishing of float-glass surfaces. This interpretation is supported by the high SiO_2_ content determined for MO, which is consistent with the presence of SiC residues.

**Figure 1:**
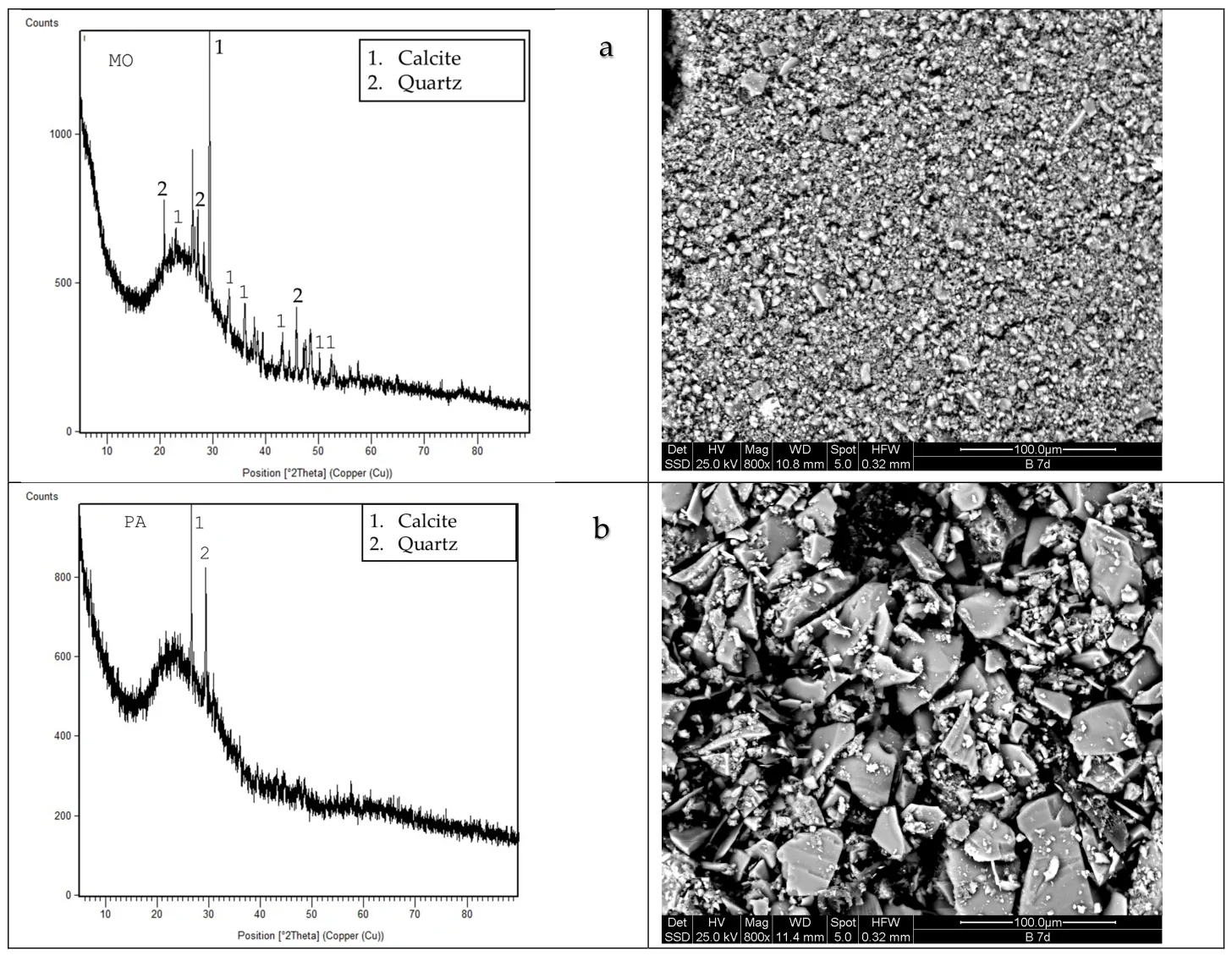

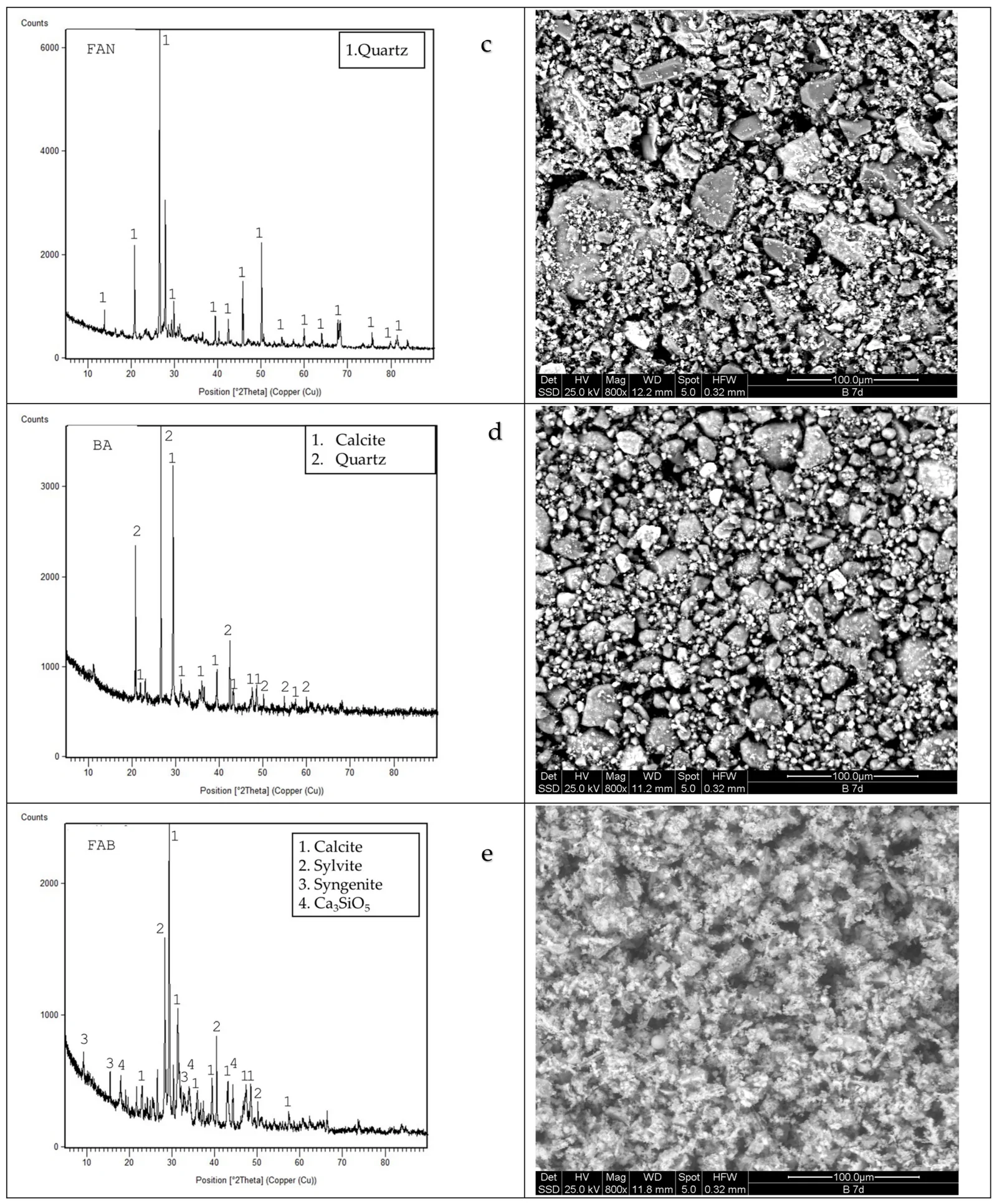
XRD patterns and SEM images of the five wastes: (**a**) Float-glass polishing sludge (MO), (**b**) fine glass dust (PA), (**c**) fly ash (black) (FAN), (**d**) bottom ash (BA), (**e**) fly ash (white) (FAB).

The mineralogical and microstructural characterization of the wastes provides essential information for understanding their reactivity (Figure 1).

Regarding the crystalline phases, a general trend can be observed: wastes originating from high-temperature processes (FAN, FAB, and BA) exhibit a higher degree of crystallinity than those derived from the mechanical processing of glass products (MO and PA). Low-quartz (SiO_2_) and calcite (CaCO_3_) crystals are the most identified crystalline phases across the investigated wastes (Figure 1). In particular, FAB shows a more complex mineralogical composition, with the presence of calcite, sylvite (KCl), calcium silicate (Ca_3_SiO_5_), and syngenite (K_2_Ca(SO_4_)_2_·H_2_O). These phases are consistent with the high concentrations of K and S, which, together with Ca, are among the most abundant elements detected in this waste. For the metakaolin XRD pattern, please see Figure S1 of the Supplementary Materials. The three crystalline phases were quartz, anatase and muscovite, as expected from the literature [13] and manufacturer data.

From a microstructural perspective (Figure 1, SEM images), it is interesting to note that, although all waste powders were sieved to below 45 μm, the particle size distributions of the passing fractions appear to differ significantly among the investigated materials. Based on the SEM observations (Figure 1), MO appears to contain the finest particles, followed by FAB, FAN, BA, and PA. Therefore, the sieving process resulted in powders with the same upper particle size limit but different degrees of fineness. Nevertheless, fine particles can be observed in all five waste materials, including PA, which may contribute to a certain extent to their reactivity. Obviously, a grinding operation could have produced higher fractions of very fine powders, and this represents an opportunity for further investigations.

Another relevant aspect common to all the powders is their homogeneity. Chemically speaking, since the SEM photos were collected using a backscattered electron detector, the gray scale of the image corresponds to the increasing atomic weight of the element contained in the particle, from dark to light gray. BA presents brighter grains which appear Fe-rich in the EDS chemical analysis. A similar observation, but to a smaller extent, can be made for FAN, again supported by EDS analyses not reported here, fully supporting the XRF results for iron content.

### 3.2. Characterization of the Alkali-Activated Binders

#### 3.2.1. Chemical and Mechanical Stability

The consolidated alkali-activated binders were visually inspected after demolding, and only specimens free from visible defects were selected for further characterization (Table 4).

The stability of the reaction products in water was evaluated as an indicator of the effective development of the alkali-activated network [19]. As discussed in Section 3.1, the relatively high CaO content of FAN, BA, and FAB is expected to promote the formation of calcium-containing reaction products. In particular, the coexistence of C-S-H and N-A-S-H gels may exert a synergistic effect on matrix consolidation, resulting in improved chemical stability and overall performance of the alkali-activated binders [25,26,27,28,29].

The first results presented in this section concern the water stability of the consolidated binders. The presence of a well-developed alkali-activated aluminosilicate network, with or without calcium-containing reaction products such as C-S-H gel, is expected to avoid sample deterioration after 24 h of immersion in distilled water. All the samples that were free from visible defects after demolding (Table 4) successfully passed the integrity test. The absence of visible degradation, as demonstrated by the clarity of the leachate and the preservation of the mechanical integrity of the specimens, indicates the good chemical stability of the alkali-activated binders (see Figure S2 of the Supplementary Materials).

A more quantitative extent of geopolymerization and network development within the N-A-S-H/C-(A)-S-H matrix was obtained from measurements of ionic conductivity and pH of the eluates collected after 24 h of immersion in water. In this specific case, a solid-to-liquid ratio of 1:10 was adopted according to the EN12457-2 standard. As evidenced by the maximum percentage deviation among replicate measurements (±2.3% for pH and ±13.4% for ionic conductivity), pH is less sensitive than ionic conductivity to changes in the concentration and nature of the ionic species released into the leachate, since it mainly reflects the concentration of OH^−^ ions.

Despite the intrinsic heterogeneity of both the waste materials and the resulting alkali-activated binders, which contributed to some variability in the measured values, clear trends can be identified as the waste content increases relative to the reference MK-based geopolymer (GP0) in both the S (substitution of MK) and R3 (addition to MK) series. Both ionic conductivity and pH exhibit an increasing trend with increasing waste incorporation and generally reach higher values when the waste is used as a partial replacement for MK rather than as an addition to the fresh reference geopolymer paste. In the former case, the alkaline activator-to-reactive precursor ratio is higher, resulting in lower consumption of the activating solution during precursor dissolution and gel formation. Consequently, a larger fraction of free alkaline species and dissolved ions remains in the pore solution, leading to higher pH and ionic conductivity values of the eluates.

Except for formulations S_PA5 and S_FAB50, all samples produced eluates with ionic conductivity values below 30 mS/cm. Despite being approximately three times higher than that measured for the reference geopolymer GP0, this conductivity level cannot be considered indicative of degradation or substantial disruption of the aluminosilicate framework based on the literature data [21]. This interpretation is further supported by the satisfactory chemical stability previously observed in dissolution tests conducted at a solid-to-liquid ratio of 1:100. Among the investigated wastes, PA- and FAN-containing formulations exhibited the lowest ionic release when substituted for MK, suggesting greater reactivity toward the alkaline activating solution and more effective incorporation into the alkali-activated matrix than the other waste types.

The value of the pH of the eluate is above 12 for formulations S_PA5 and S_FAB50, indicating that all the others do not have a large excess of unreacted alkaline solution. Most of the formulations present a pH value below 11, which indicates the absence of an excess of unreacted alkaline solution [37,38].

**Figure 2:**
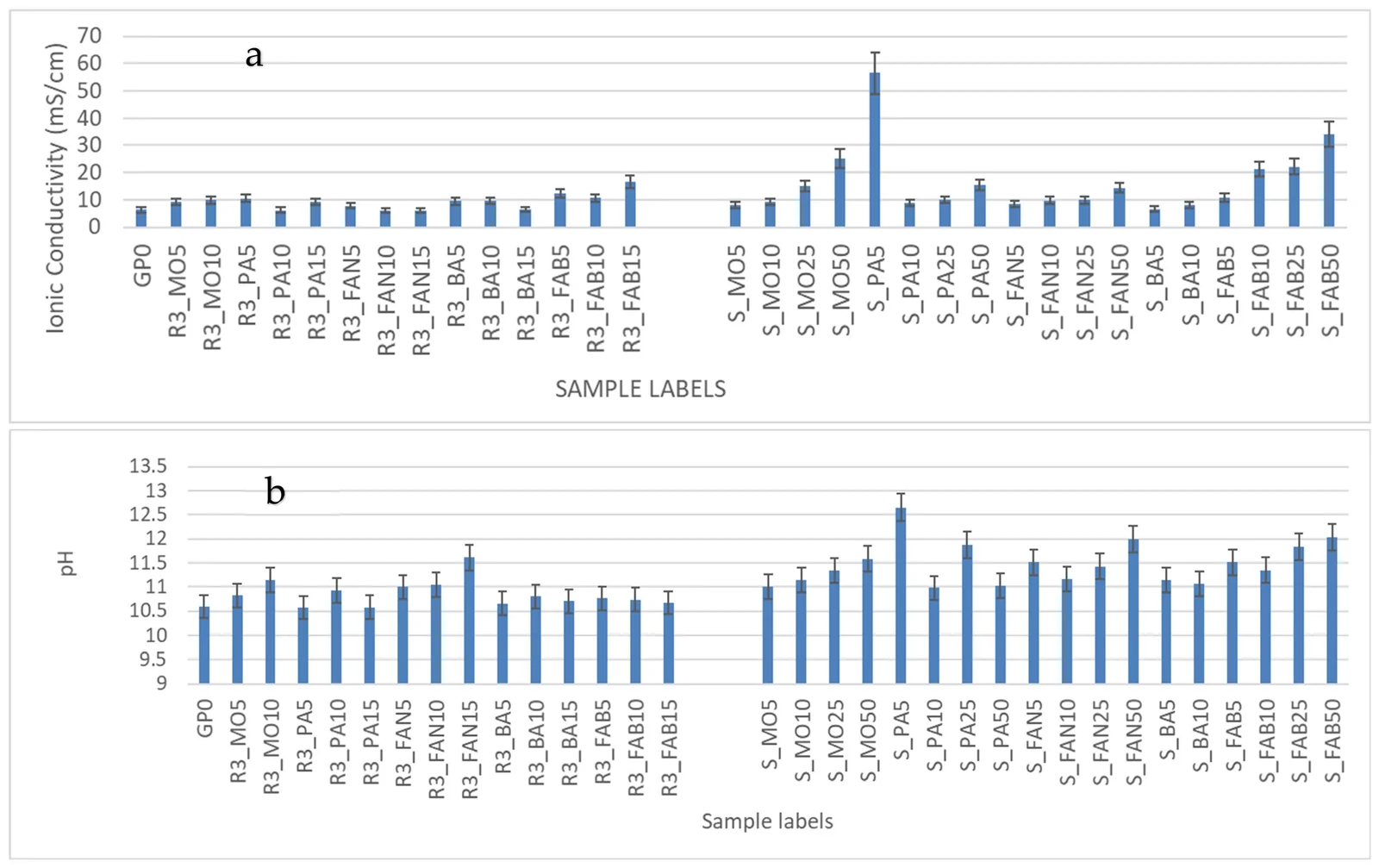
Results of (**a**) ionic conductivity and (**b**) pH collected on the eluate after immersion of the solid piece of each different composition (solid/liquid = 1:10, 24 h). The reported error corresponds to the maximum percentage deviation among the measured values: ±2.3 % for pH and ±13.4 % for ionic conductivity.

The consolidated alkali-activated binders listed in Figure 2 and presenting optimal chemical stability were also tested for compression resistance, with the aim of proposing a faster evaluation test to individuate the performance of the addition or substitution of a certain waste in a geopolymer matrix.

It should be remembered that the percentage of addition of MO could not exceed 10%; otherwise, the workability would be penalized. This is the reason why formulation R3_MO15 is not presented in Table 4, where the results of the compressive strength are reported together with the apparent density and comments on the defects (see also images of defects in Figure S3 of the Supplementary Materials). It should be noted that the compressive load was applied until the load indicator showed that the load was decreasing steadily and the specimen displayed well-defined fracture patterns defined as Types 2 and 3 by ASTM C39 [22]. Images of fracture geometries are reported in Figure S4 of the Supplementary Materials.

The compressive strength values reported in Table 4 are corrected according to ASTM C39 [22] when the specimen length-to-diameter ratio was less than 2.0. Uncorrected compressive strength data are reported in Table S2 of the Supplementary Materials.

The reference geopolymer (GP0), composed of 100% metakaolin, serves as the mechanical baseline with a compressive strength of 16.2 MPa. The compressive strength of the reference MK-based geopolymer was relatively modest with respect to the literature data [39,40]; however, this formulation was adopted as the benchmark owing to its excellent reproducibility and processing reliability. Its high fluidity ensured the effective incorporation and homogeneous dispersion of the dry waste powders in the addition-based mixtures, thereby minimizing variability associated with mixing and casting procedures [13]. Nevertheless, the same rheological characteristics were accompanied by a lower compressive strength when the material was tested as a neat alkali-activated binder rather than as a mortar, where the presence of aggregates generally contributes to improved mechanical performance [41].

As a general trend, waste substitution (S-series formulations) resulted in higher compressive strengths than waste addition (R3-series). This behavior can be attributed to the greater availability of alkaline activator in the substituted formulations, which likely promoted more extensive dissolution of the precursor phases, enhanced geopolymerization, and ultimately the development of a denser and more interconnected reaction network [39].

The role of the investigated wastes extended beyond that of an inert fine aggregate, as demonstrated by comparison with the formulation containing sand addition (Figure S5 in the Supplementary Materials and comments in Section 4). Among the substituted mixtures, float-glass polishing sludge (MO) and black fly ash (FAN) exhibited the most beneficial effects on mechanical performance, both reaching maximum compressive strengths at a substitution level of 10 wt%. However, FAN showed a particularly high reactivity, maintaining satisfactory strength values even at replacement levels as high as 50 wt%. The synergetic effect of fly ash addition to metakaolin is a very well-known phenomenon [42,43]. White fly ash (FAB) also acted as an effective reactive component, leading to compressive strengths higher than those of the reference formulation and reaching maximum performance at substitution levels between 10 and 25 wt%. Fine glass dust (PA) exhibited a similar behavior, although a moderate strength reduction was observed at the highest substitution level (50 wt%) [31]. In contrast, bottom ash (BA) showed limited reactivity under the investigated conditions. The corresponding formulations failed to reach the compressive strength of the metakaolin-based reference binder at any replacement level, and specimens containing 25 wt% BA or more were unable to develop sufficient cohesion to produce self-supporting monolithic materials. In this last case, a proper formulation of the binder mix is required [27].

Similar trends were observed when the waste materials were added as dry powders to the fresh GP0 geopolymer paste, with MO again exhibiting the highest mechanical performance at an addition level of 10 wt%. The beneficial effect of MO can be attributed to its dual function within the alkali-activated matrix. Owing to its fine particle size and low porosity, MO contributes to matrix densification through a filler effect, while simultaneously acting as a partially reactive component capable of interacting with the alkaline environment [41]. The combined action of these physical and chemical contributions is likely responsible for the observed enhancement in compressive strength.

Apparent density values do not vary so much, ranging from 1.24 to 1.51 g/cm^3^, and did not follow the commonly observed rule whereby higher density corresponds to higher mechanical strength. They show a trend of increasing with the percentage of each single waste, but this trend is not strong. We can assume that some pores might affect single values of density as well as strength, even though the trends are still evident [44]. Nevertheless, these values of density assure proper application of the mechanical assessment according to ASTM C39 [22].

**Figure 3:**
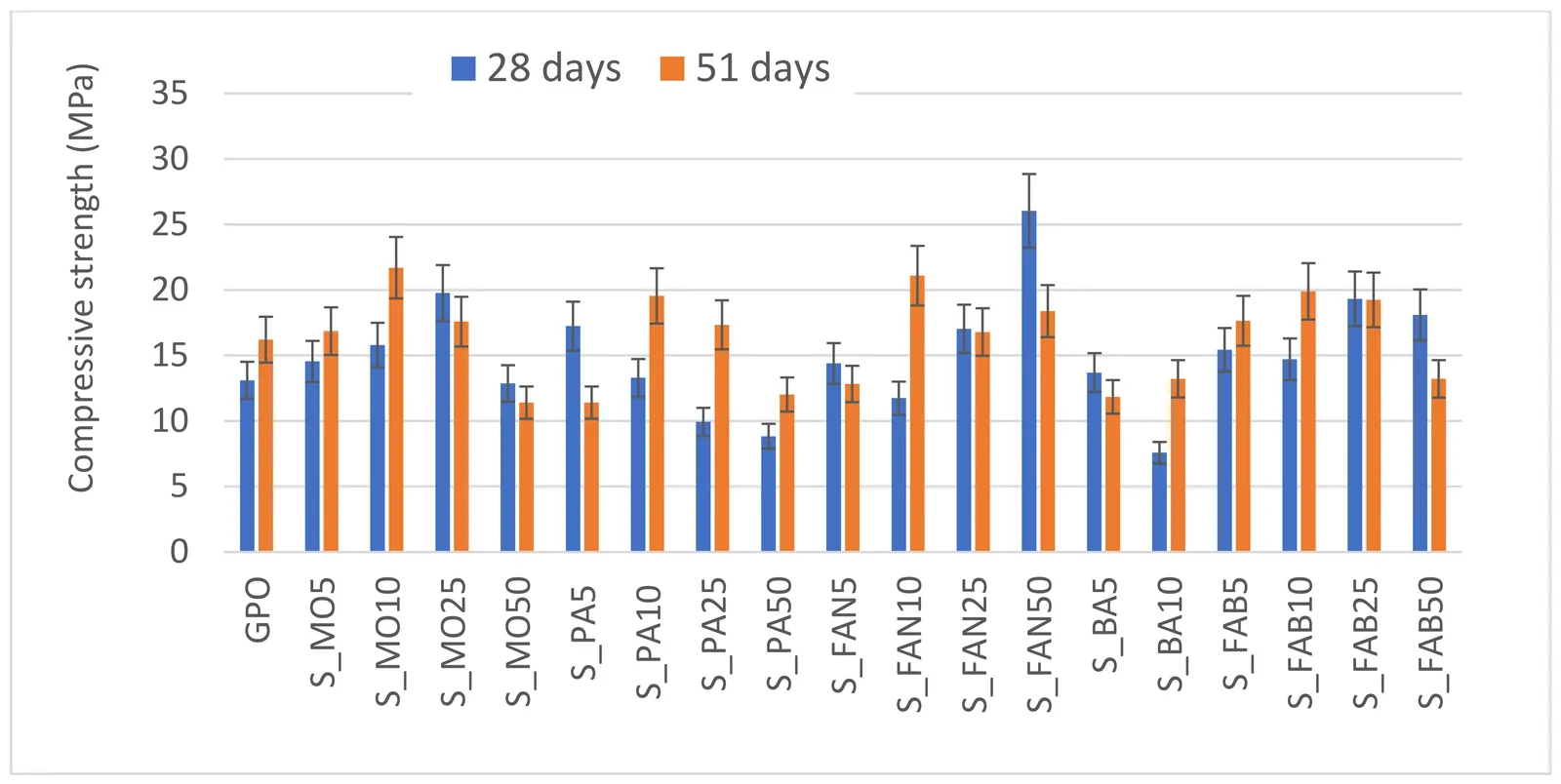
Compressive strength results corrected for geometric factor of samples with wastes substituted for MK after 28 and 51 days of curing. The error bar corresponds to the maximum percentage deviation of ±10.8%.

The effect of curing time was also tested exclusively for the substitution-based formulations. These mixtures were considered more relevant than the addition-based systems, as the primary objective of this study was to assess the reactivity of the waste powders in an alkaline environment rather than their contribution as inert fillers or fine aggregates. Figure 3 compares the compressive strength measured after 28 and 51 days of curing. Extending the curing period to 51 days resulted in a further increase in compressive strength for all investigated wastes, with the exception of MO and FAN, for which no significant improvement was observed.

#### 3.2.2. Reactivity Degree and Microstructural Characterization

Further investigation on the reactivity of the formulations exhibiting the highest mechanical performance for each waste is presented in this section.

**Figure 4:**
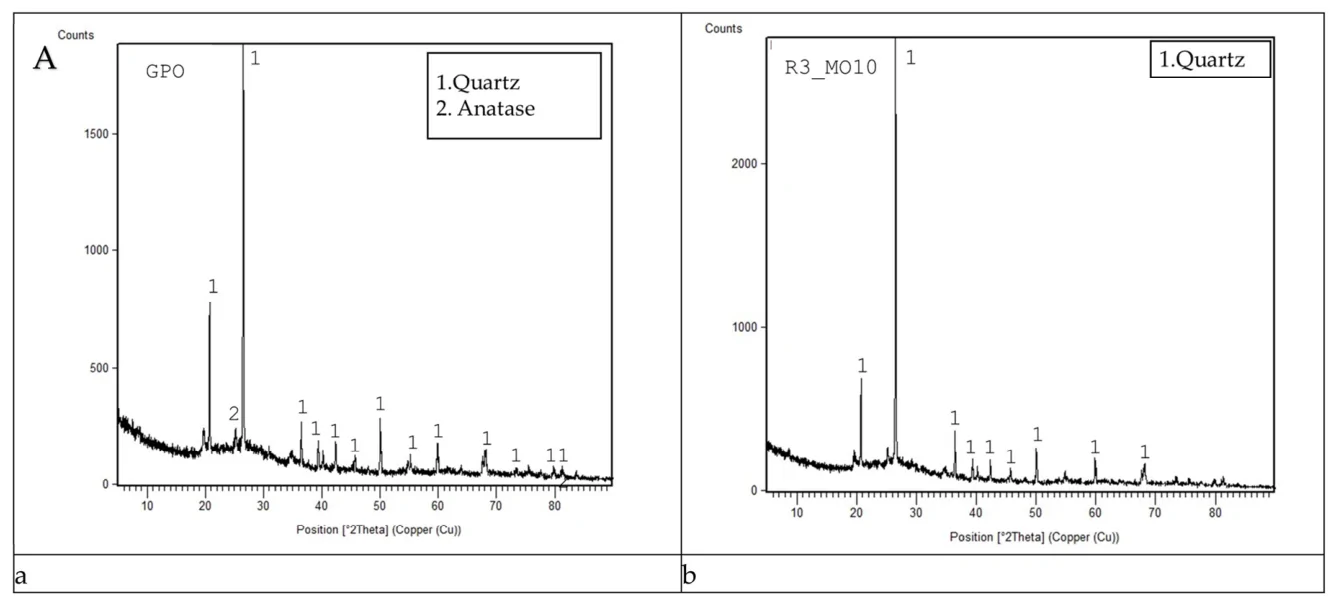

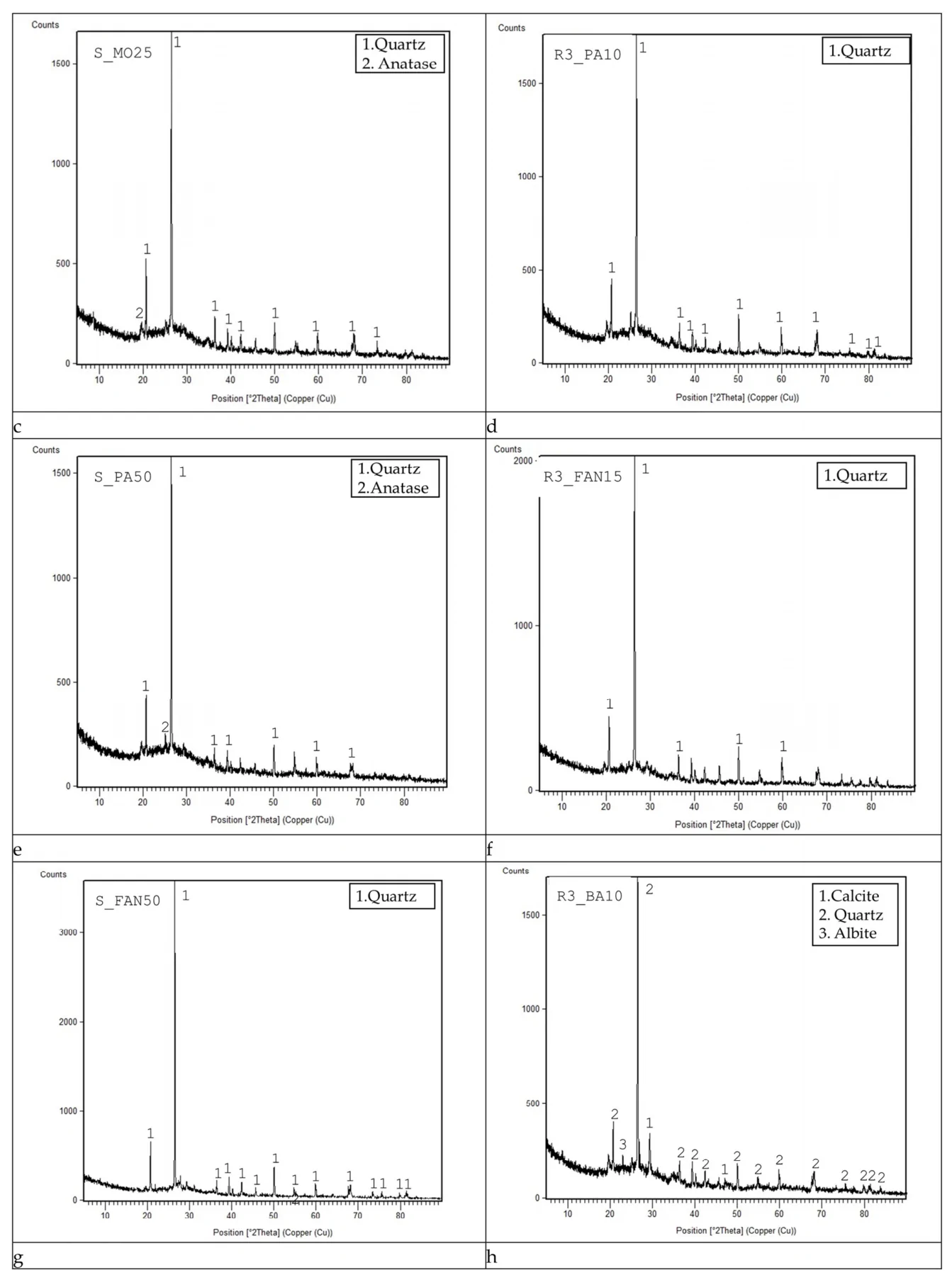

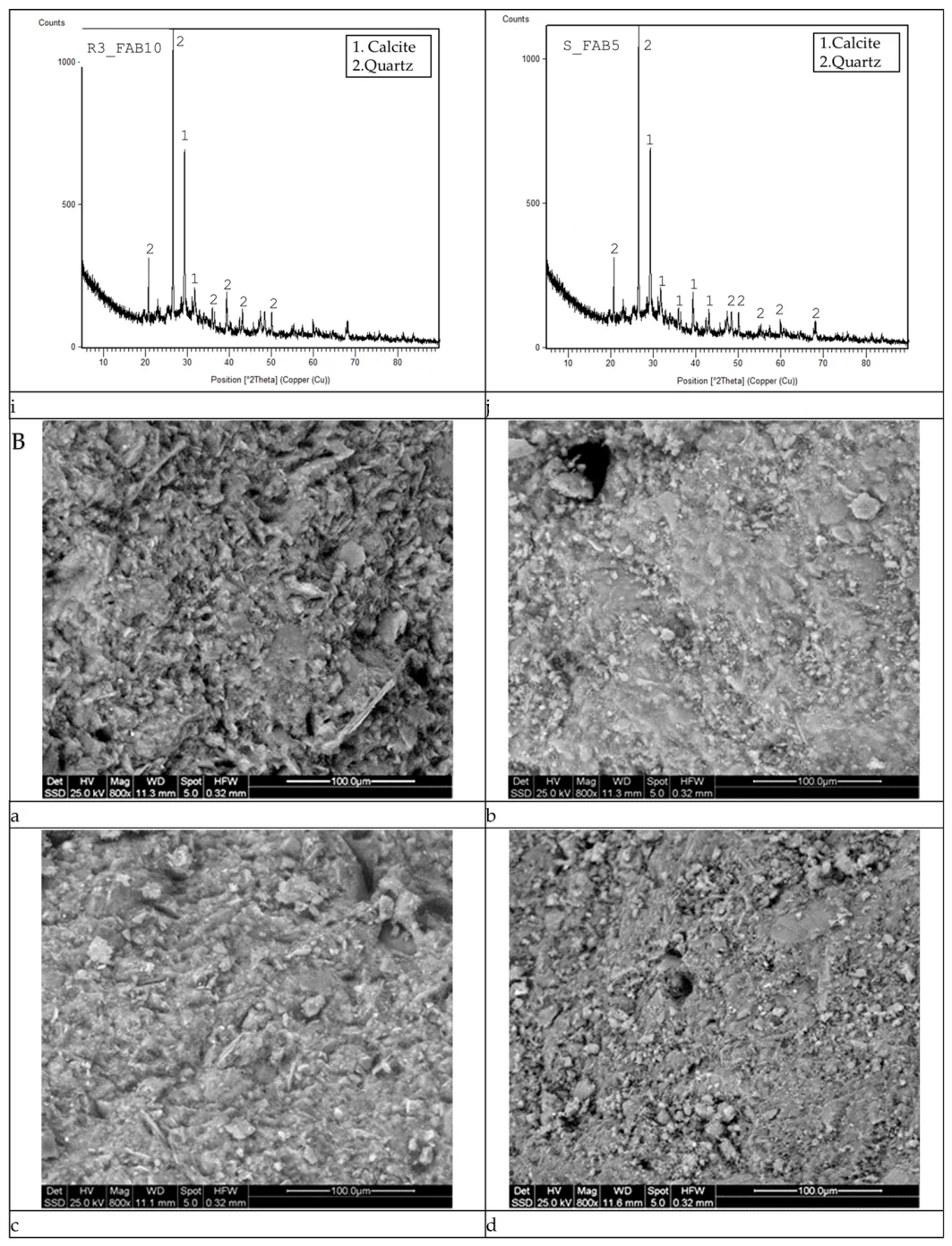

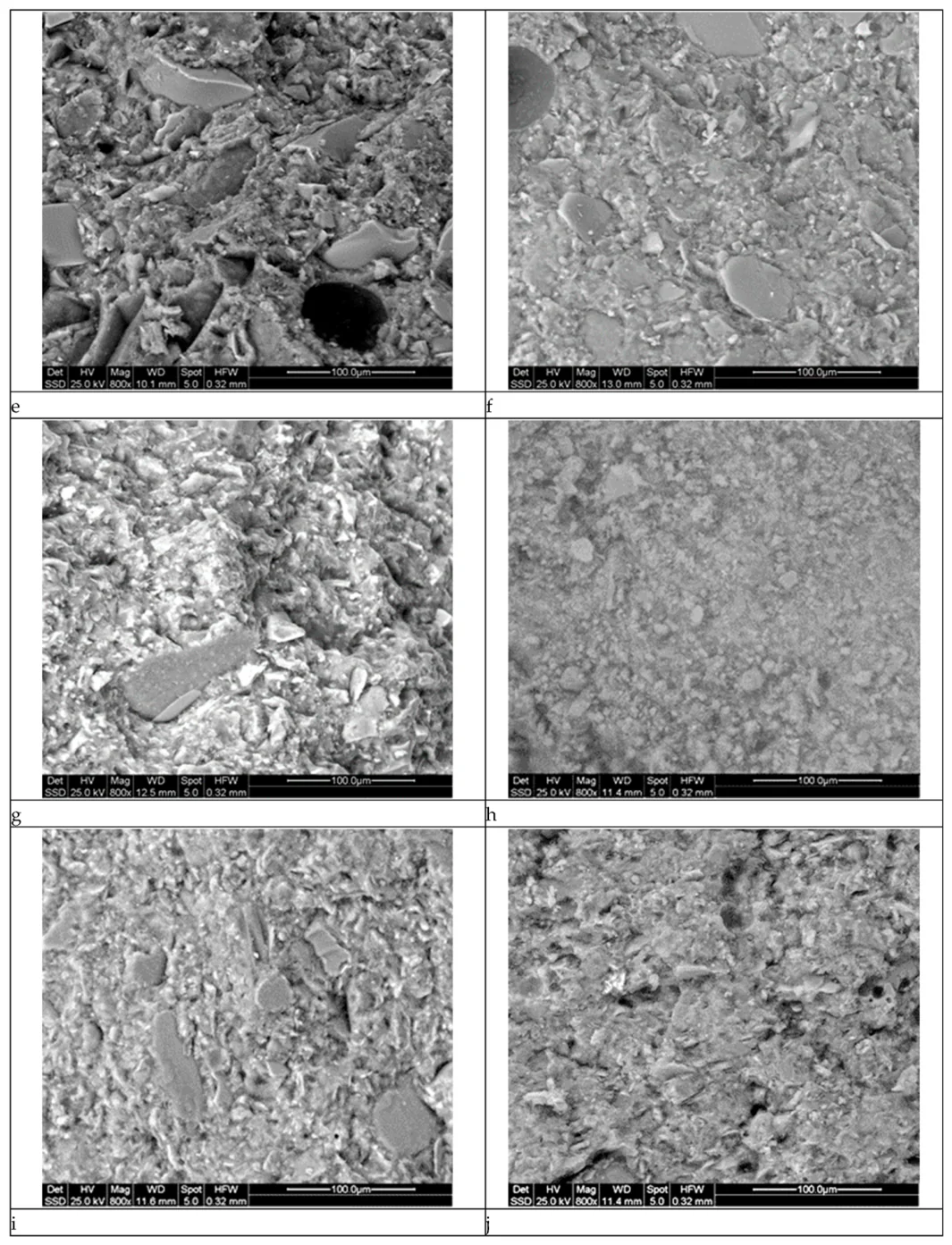
(**A**) XRD patterns and (**B**) SEM images (800×) of the alkali-activated binders with the highest mechanical strength and the reference formulation: (**a**) GP0, (**b**) R3_MO10, (**c**) S_MO25, (**d**) R3_PA10, (**e**) S_PA50, (**f**) R3_FAN15, (**g**) S_FAN50, (**h**) R3_BA10, (**i**) R3_FAB10, (**j**) S_FAB5.

In Figure 4, XRD patterns highlighted the presence of several crystalline phases within the alkali-activated binder’s matrix, specifically calcite, quartz, and silicon oxides. These phases were the same as those identified in the different wastes (Figure 1) and no new crystalline phase was evidenced. A broad band typical of the amorphous geopolymer gel is present in all the investigated samples, hindering the peaks of the crystalline phases present in very small amounts [45]. The only phases which disappeared (totally or partially except for the overlap with the amorphous band) are those in FAB materials, i.e., sylvite, calcium silicate and syngenite. In particular, the disappearance of the peaks attributed to calcium silicate is indicative of the formation of a Ca-rich gel phase in the solidified binders.

SEM micrographs revealed the development of highly compact matrices, particularly in the formulations containing MO, FAN, and FAB, where only residual grains of the original waste materials remained distinguishable. The high compressive strengths measured for these binders are consistent with the observed microstructural densification. In these samples, the waste particles appear to be partially encapsulated by geopolymer [46]. Moreover, the interfaces between the alkali-activated matrix and the embedded particles appear dense and free from visible microcracks or debonding features, indicating good interfacial adhesion and efficient stress transfer within the composite material [47]. EDS analyses were performed at high magnification (3000×) on the hardened binders containing the investigated wastes, both in replacement and addition formulations. However, these analyses did not allow a clear distinction between the N-A-S-H and C-S-H gel phases. In samples containing significant amounts of Ca-bearing phases (FAN, FAB, and BA), the measured compositions were consistent with the presence of N/C-A-S-H gels, whereas N-A-S-H gel compositions were identified in the formulations containing MO and PA. No discrete C-S-H phase could be unequivocally identified (Figures S6–S8 of the Supplementary Materials).

**Figure 5:**
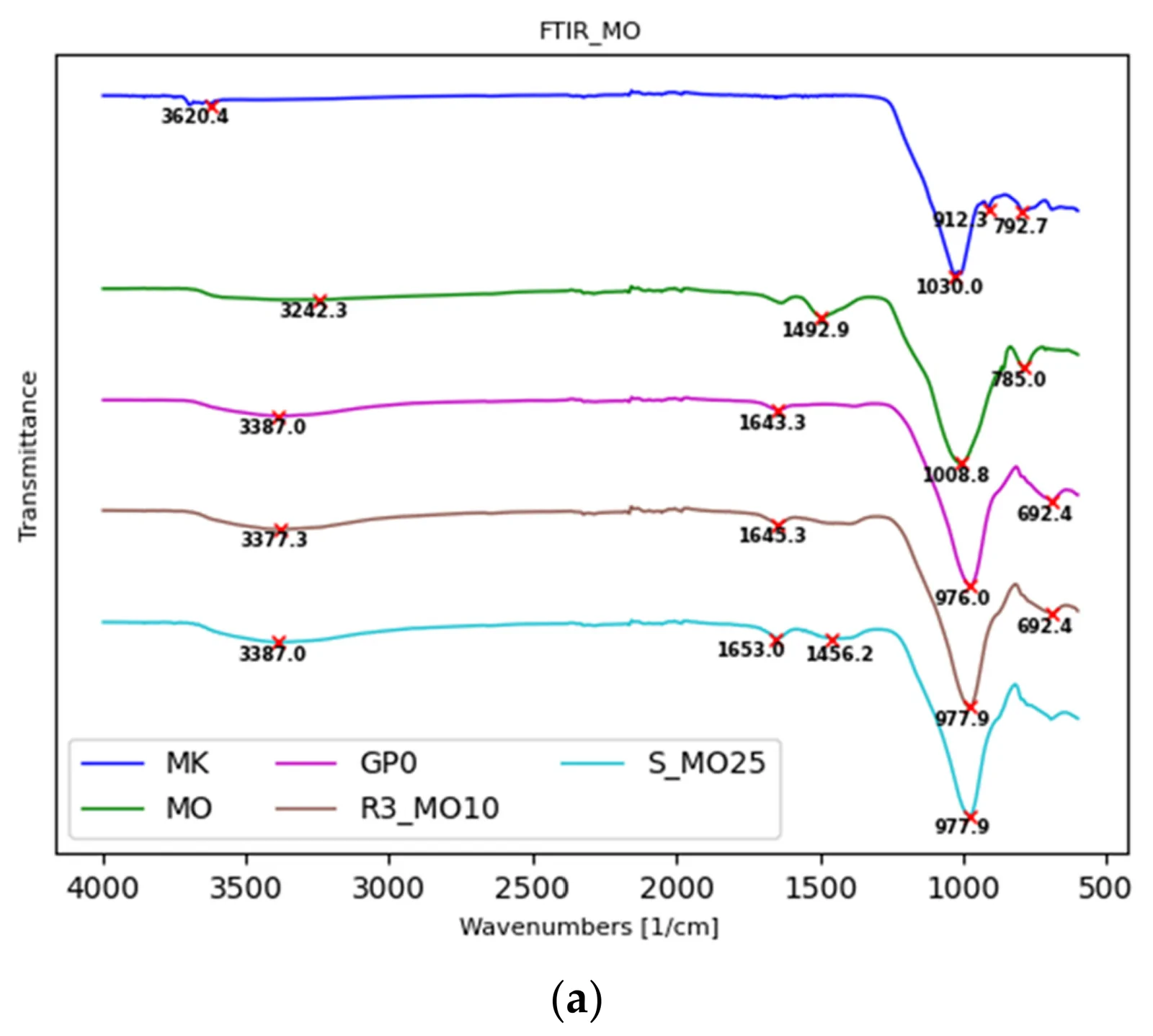

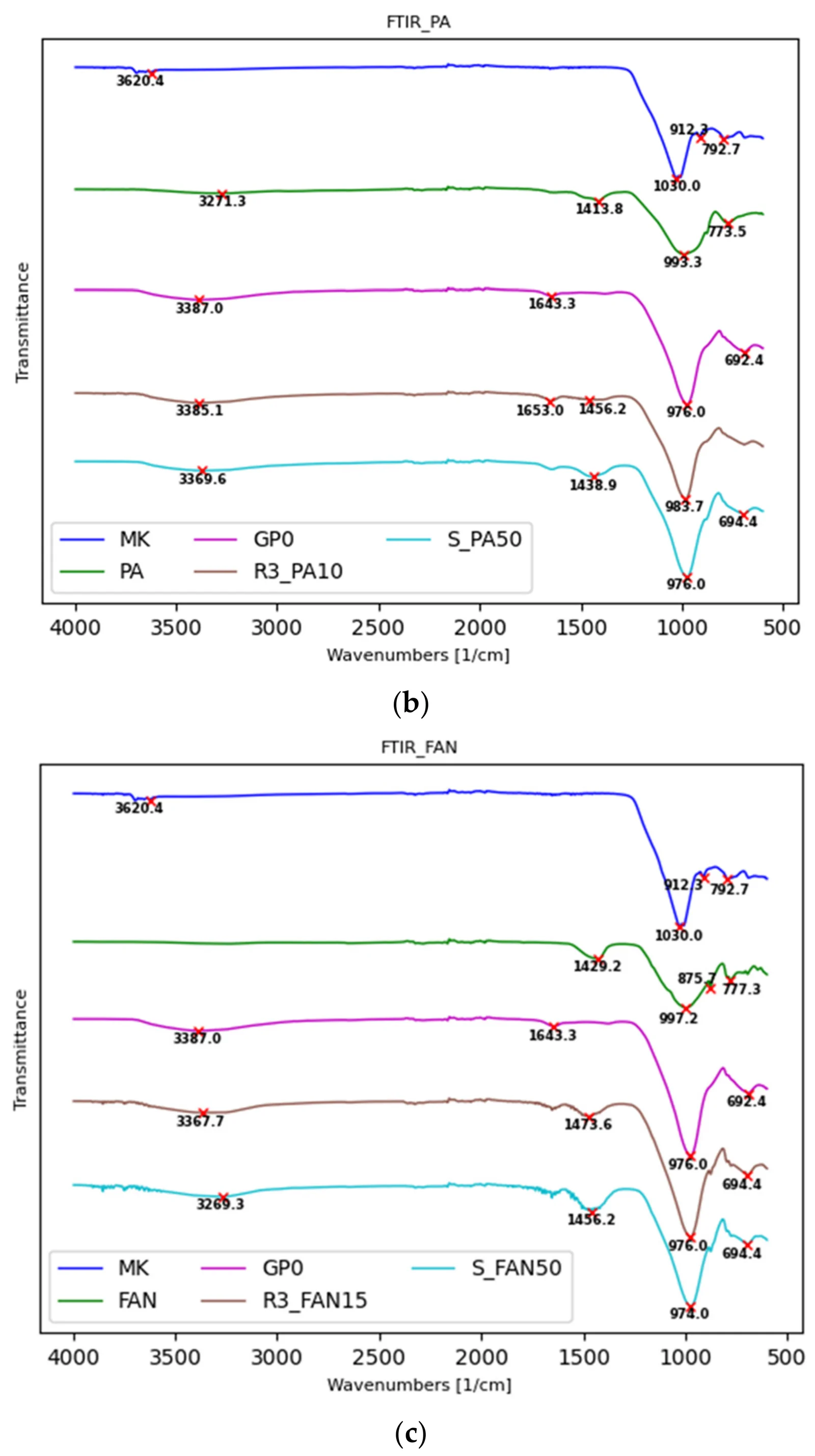

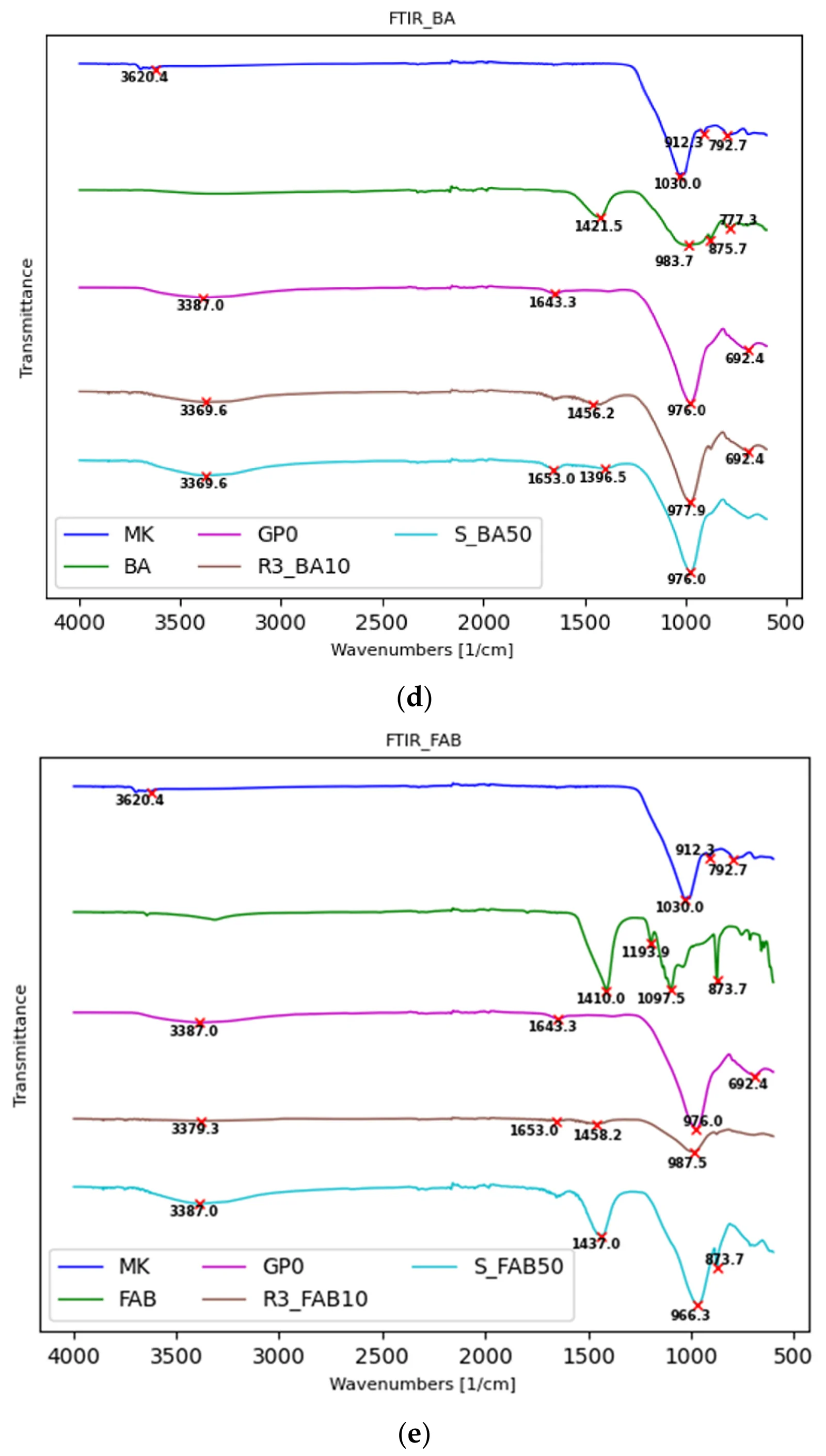
FT-IR patterns of the alkali-activated binders with optimal mechanical performance and reported with respect to metakaolin (MK), the reference formulation GP0, and the waste for: (**a**) R3_MO10 and S_MO25, (**b**) R3_PA10, and S_PA50, (**c**) R3_FAN15 and S_FAN50, (**d**) R3_BA10 and S_BA50, and (**e**) R3_FAB10 and S_FAB50.

Figure 5 shows the FT-IR spectra collected on the samples with higher mechanical resistance. The typical asymmetric stretching of the Si-O-T (T=Si or Al) bond appears as the most pronounced peak around 1000 cm^−1^, and the metakaolin is shifted to lower wavelength numbers after the geopolymerization [48]. This shift is interpreted as a consequence of Al penetration into the original structure of the Si-O-Si skeleton and it is considered indicative of the formation of the geopolymer gel [49,50,51,52]. This peak remains almost unaltered by the substitution of the waste for metakaolin and even in the case of its addition.

The second most intense absorption band, centered at approximately 1450 cm^−1^, was detected in most of the investigated wastes and in the corresponding alkali-activated binders. This band is attributed to the asymmetric stretching vibration of carbonate groups (CO_3_^2−^) [53]. Its persistence after alkali activation is more likely associated with residual carbonate-bearing phases inherited from the waste precursors than with the formation of sodium carbonate through the reaction of excess alkalis with atmospheric CO_2_. This interpretation is supported by the absence of efflorescence on the surface of the specimens, even after prolonged curing (28–51 days), and by the lack of carbonate crystalline deposits in the SEM micrographs. Therefore, the carbonate-related FT-IR signal may be regarded as evidence that a fraction of the original waste particles remained only partially reactive during the alkali activation process.

The wide bands with low intensities observed at 1640 and 3440 cm^−1^ are attributed to the stretching vibration of the O-H bond and the bending vibration of the O-H-O bond, respectively [54,55]. Their scarce presence indicates a reticulation of the Si-O-H groups to Si-O-Si ones.

These results underline again the stability of the geopolymer network and the good level of 3D reticulation.

## 4. Discussion

The present study was conceived to comparatively evaluate the reactivity or “cementing activity” and potential valorization of five industrial wastes singularly integrated within an alkali-activated MK-based system using a single mechanical compressive strength. To this end, a highly reproducible and processable MK-based geopolymer (GP0) was selected as the benchmark formulation [10,11,12,13]. Its relatively high fluidity ensured the effective incorporation and homogeneous dispersion of dry waste powders in the addition-based mixtures, thereby limiting variability associated with mixing and specimen preparation. While the high liquid content required to achieve such workability resulted in a comparatively low compressive strength when GP0 was tested as a neat binder, this limitation was accepted in order to establish a robust and reproducible reference system for assessing the contribution of each waste.

The lack of rheological data (such as flowability, viscosity, etc.) is here substituted with a qualitative workability assessment. As this study reports a preliminary investigation, we acknowledge that this aspect could be further refined and explored in future work. By adopting the same range of particle size for all the investigated wastes and fixing the maximum replacement level at 50 wt% of a commercial metakaolin, we believe that the reproducibility of the proposed testing approach across different waste materials can be reasonably ensured.

**Figure 6:**
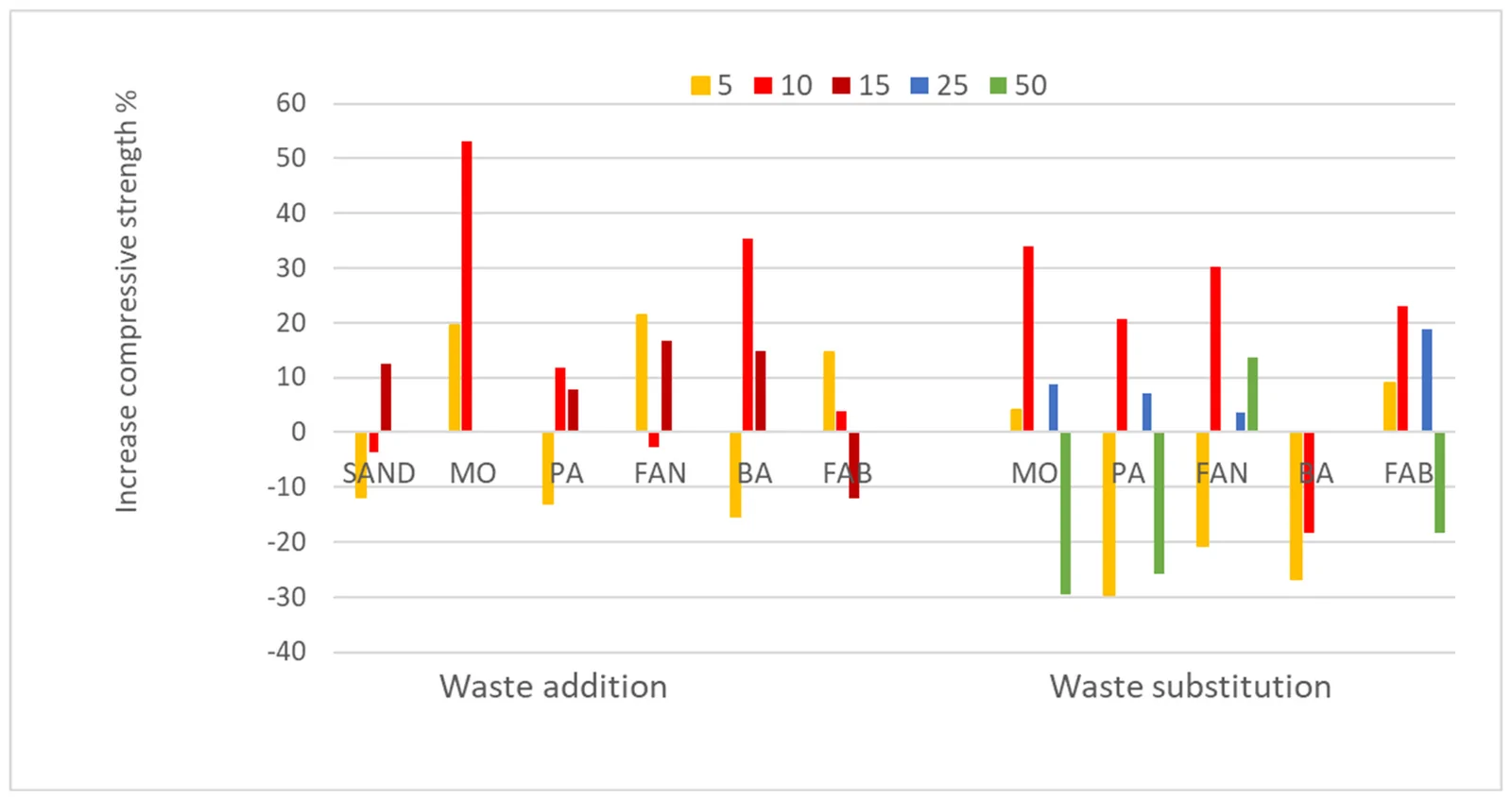
Variation (in %) in the compressive strength of the alkali-activated binders added or substituted with single wastes (MO, PA, FAN, BA, FAB) with respect to the reference formulation GP0 with sand addition (SAND).

Within this experimental framework, remarkable improvements in mechanical performance were observed following waste incorporation (Figure 6). Experimental data for mechanical performance are discussed as comparative values, rather than absolute values, since real optimization of the binder mix is not presented in this study. In order to accurately quantify the effect of each waste on the compressive strength of the alkali-activated binders, the influence of specimen geometry was taken into account following the recommendations of ASTM C39. Compressive strength measurements are known to be affected by the height-to-diameter ratio (H/D), with lower H/D values generally resulting in higher apparent strengths due to the confinement effect induced by friction between the specimen ends and the loading platens. Consequently, shorter cylinders may yield artificially inflated strength values when compared with standard specimens with H/D = 2. To eliminate this geometrical bias, the measured strengths were corrected using the appropriate shape factors before data analysis. The resulting normalized values (Table 4) allowed the effect of waste addition or substitution to be evaluated on a consistent basis and directly compared with the benchmark GP0 formulation, as illustrated in Figure 6. A maximum curing time of 51 days was adopted for all the formulations, even though a very similar trend could be found for samples aged 28 days (with data reported in Figure 3). A heatmap with numerical annotations (Figure S9 of the Supplementary Materials) provides both a quantitative representation of the trends and a concise overview of the entire dataset.

Compressive strength increased significantly for formulations containing float-glass polishing sludge (MO), black fly ash (FAN), and white fly ash (FAB) additions, with the most pronounced enhancements recorded at waste contents between 10 and 15 wt%. Moreover, FAN exhibited a particularly beneficial effect, maintaining a positive contribution even when used as a replacement for MK at levels up to 50 wt%. These results highlight the high reactivity of these waste streams and their potential to act not only as fillers but also as active constituents in the development of alkali-activated binders.

Similar findings have been reported in the literature for comparable fly ash-containing systems, where the extent of geopolymerization was assessed through semi-quantitative XRD analysis [56]. Further investigations in this direction would be valuable, particularly for the most promising waste streams identified in this study, namely FAN, MO, and FAB.

The observed mechanical performance indicates that these materials do not act simply as inert fillers, but actively participate in the reaction process through their interaction with the alkaline silicate activator and their contribution to gel formation. According to the powder fineness taken as a measure of physical filling effects, the sequence should have been: MO followed by FAB, FAN, BA, and PA. These findings highlight the high reactivity and binding potential of fly ashes (FAN and FAB) [29], polishing sludge, and fine glass dust (PA) in alkali-activated systems. Additionally, we can comment that with respect to the sand addition, where at least 15% is required to enhance the compressive strength, other wastes have favorable effects already at 10 wt% in addition and/or in substitution.

For most formulations, the observed improvement in compressive strength was accompanied by limited ionic leaching and only marginal changes in the FT-IR spectra after waste incorporation. These findings indicate that the waste materials contributed positively to the development of the alkali-activated matrix while preserving the fundamental molecular structure of the reaction products. The reactivity of the waste with the alkaline environment was evident even at replacement levels of up to 50 wt% of metakaolin, with FAN showing particularly beneficial effects and, in some cases, superior mechanical performance compared with the reference formulation. In contrast, waste addition to the fresh geopolymer paste was associated with a higher solid-to-liquid ratio, which adversely affected workability, limited proper particle dispersion, and reduced the degree of matrix homogenization.

Unfortunately, many practices still conceive industrial inorganic wastes as inert aggregates and filler materials, which slows down their valorization [57,58]. The presence of calcite and quartz phases identified in our XRD analysis contributed to microstructural densification. Calcium-rich phases can coexist with geopolymer gels, and this synergy creates a more robust and mature 3D network. In contrast, bottom ash (BA) consistently failed to meet the performance of the pure metakaolin reference due to its highly crystalline nature, which restricted the availability of reactive aluminosilicates during activation [1]. Such failures are due to an excessive silica-to-alumina ratio [59]. Higher silica content can create linear, rubbery structures rather than rigid 3D networks [1].

Our study fills a critical gap identified in [60], whose author’s parametrization models often focus on high-purity precursors. We extended this approach by resolving the impact of heterogeneous mechanical substitutions. Similarly, the primary data we provide for metakaolin-based systems addresses the “fly ash-centric” bias found in meta-analyses [61]. The chemical variability of industrial by-products remains an unresolved challenge. However, this variability can also be seen as an opportunity, particularly in the context of urban mining [62]. Our data helps categorize diverse waste streams into specific reactivity classes. This methodology has been proposed to move toward chemistry-based standardization [63]. We have established a solid baseline for the sustainable valorization of local industrial residues, and this foundational case study contributes to the development of future performance-based design standards.

## 5. Conclusions

This study demonstrates that substituting metakaolin with inorganic non-hazardous industrial wastes can significantly enhance the mechanical performance of geopolymer binders. Black fly ash (FAN) emerged as the most effective precursor at high waste content, increasing the compressive strength by 1 % at 50 wt% substitution with no mix design optimization. White fly ash (FAB) and float-glass polishing sludge (MO) also proved to be stable, high-efficiency binders. Conversely, the use of bottom ash (BA) and high concentrations of fine glass dust (PA) led to mechanical declines due to high crystallinity and unfavorable silica-to-alumina ratios or limited reactivity of the amorphous fraction. Microstructural analysis confirms that the synergy between geopolymer gels and crystalline phases like calcite or reactive Ca-bearing phases and quartz facilitates matrix densification.

A proper reformulation could improve workability, reactivity and mechanical and chemo-physical performance. Reformulation was not the objective of this investigation, but in the future we will dedicate time to the optimization and upcycling of these five wastes.

Ultimately, these findings establish a baseline for the sustainable valorization of local industrial residues, contributing to the development of chemistry-based standardization for future green infrastructure.

## Figures and Tables

**Table 1:** Mix design of geopolymer formulations prepared by partial replacement of metakaolin (MK) with individual waste materials (S Series). The reference formulation (GP0) corresponds to 0 wt% MK replacement. Percentages refer to the amount of dry MK powder replaced.

Percentage of Substitution	Metakaolin (wt%)	Waste (wt%)	Activator (wt%)
0%	56.18	-	43.82
5%	53.37	2.81	43.82
10%	50.56	5.62	43.82
25%	42.14	14.04	43.82
50%	28.09	28.09	43.82

**Table 2:** Mix design of geopolymer formulations prepared by the addition of individual waste materials or standardized sand to the fresh paste of the reference formulation GP0 (R3 Series). Percentages refer to fresh paste mass.

Percentage of Addition	Metakaolin (wt%)	Waste or Sand (wt%)	Activator (wt%)
5%	53.52	4.76	41.72
10%	51.07	9.09	39.84
15%	56.32	13.04	30.64

**Table 3:** Major chemical composition of the five waste materials, as determined by XRF and expressed as oxide content (wt%). Elements grouped under *Others* include Mn, La, Zn, Cu, Fe, Ce, Cl, and Ti. Loss on ignition (LOI) was determined after heating the powder at 1100 °C for 2 h (see also Table S1 of the Supplementary Materials). Relative accuracy for XRF data of approx. ±5–10%.

Oxides (wt%)	SiO_2_	Al_2_O_3_	CaO	Fe_2_O_3_	MgO	Na_2_O	K_2_O	P_2_O_5_	SO_3_	Others	LOI
Float-glass polishing sludge (MO)	63	1.4	7.9	-	3.8	7.9	-	-	-	0.3	15.2
Fine glass dust (PA)	61	2.9	11	1.4	3.0	10	-	-	-	2.1	8.35
Fly ash (black) (FAN)	47	10	18	4.1	0.1	1.65	8.1	1.9	1.5	3.5	2.74
Bottom ash (BA)	23	8.3	28	15	2.4	2.31	1.1	2.0	2.6	1.7	10.93
Fly ash (white) (FAB)	6.2	2.4	34	1.7	4.8	1.82	11	3.3	12	3.3	18.33

**Table 4:** Compressive strength (corrected for geometric factor) results together with density and comments on defects. Test were done on samples cured for 51 days. See also images of defects in Figure S3 of the Supplementary Materials and fracture geometries in Figure S4 of the Supplementary Materials. The maximum percentage deviation is about ±10.8% for substituted formulations, while it increases to ±18.6% for the samples with addition of wastes.

Name	Strength (MPa)	Apparent Density (g/cm^3^)	Defects
GP0	16.2	1.40	None
S_PA5	11.4	1.38	None
S_PA10	19.5	1.42	None
S_PA25	17.3	1.32	None
S_PA50	12.0	1.26	Limited efflorescence
S-PA100	-	-	Deformation
S_BA5	11.8	1.24	Slight porosity increase
S_BA10	13.2	1.30	Slight porosity increase
S_BA25	-	-	Swelling
S_BA50	-	-	Swelling
S_BA100	-	-	Efflorescence and swelling
S_FAN5	12.8	1.36	None
S_FAN10	21.1	1.37	None
S_FAN25	16.8	1.40	None
S_FAN50	18.4	1.48	None
S_FAN100	-	-	Cracking
S_FAB5	17.7	1.38	None
S_FAB10	19.9	1.43	None
S_FAB25	19.2	1.40	None
S_FAB50	13.2	1.49	None
S_MO5	16.9	1.34	None
S_MO10	21.7	1.40	None
S_MO25	17.6	1.40	None
S_MO50	11.4	1.51	None
S_MO100	-	-	Cracks and efflorescence
R3_PA5	14.1	1.41	None
R3_PA10	18.1	1.36	None
R3_PA15	17.4	1.04	None
R3_FAB5	18.5	1.34	None
R3_FAB10	16.8	1.43	None
R3_FAB15	14.2	1.46	None
R3_MO5	19.4	1.43	None
R3_MO10	24.8	1.37	None
R3_BA5	13.7	1.24	None
R3_BA10	21.9	1.30	None
R3_BA15	18.6	1.25	None
R3_FAN5	19.6	1.42	None
R3_FAN10	15.8	1.36	None
R3_FAN15	18.9	1.48	None

## Data Availability

The original contributions presented in this study are included in the article/Supplementary Materials. Further inquiries can be directed to the corresponding authors.

## Supplementary Materials

The following supporting information can be downloaded at: https://www.mdpi.com/article/10.3390/ma19183900/s1. Table S1. Complete chemical composition of the five wastes as obtained from XRF results expressed in oxides weight % or in parts per million (ppm). The Loss on Ignition (LOI) was determined as mass loss after leaving the powder in oven 2 h at 1100°C. Table S2: Compressive strength results completed with density and comments on defects. Tests were done on samples cured for 51 days.Figure S1: XRD pattern of pure metakaolin ARGICAL 1000. Figure S2: Example of alkali activated binder (R3-FAB15) after the integrity test: clear water and mechanical resistance of the solid indicate a good chemical stability of the geopolymers solid. Figure S3: Appearance of the formulation which were discarded due to defects: (a) Swelling observed for S_BA25, (b) Swelling observed for S_BA50, (c) large cracks observed and total rupture of the geopolymer for S_FAN100, (d) cracks and efflorescence for S_MO100, (e) deformation for S_PA100, (f) efflorescence for S_PA50, (g) and (h) efflorescence for S_BA100. Figure S4: Fracture geometries compared to fracture pattern/type reported in ASTM C39. Figure S5: Effect of addition of standard sand on the mechanical strength of the reference GP0 formulation. The tests were performed after 28 days of curing. Figure S6: SEM image and chemical composition of the gel matrix obtained by EDS spectrum elaboration for sample R3-BA10. Figure S7: SEM image and chemical composition of the gel matrix obtained by EDS spectrum elaboration for sample R3-FAN15. Figure S8: SEM image and chemical composition of the gel matrix obtained by EDS spectrum elaboration for sample S-FAN50. Figure S9: Heatmap with numerical annotations illustrating the percentage increase or decrease in compressive strength of alkali-activated binders containing waste additions relative to the reference formulation GP0 with sand addition, enabling both trend identification and rapid comparison across the entire dataset.
