# Supplementary material for: Reactivity Assessment of Diverse Aluminosilicate Wastes in Metakaolin-Based Alkali-Activated Binders
Source: Materials (Basel). 2026 Sep 14;19(18):3900. doi: 10.3390/ma19183900 (PMC13608157; doi:10.3390/ma19183900)
Supplement: Supplementary file 1 [file materials-19-03900-s001.zip › materials-4506200-supplementary.pdf]

materials

MDPI

Article

Reactivity Assessment of Diverse Aluminosilicate Wastes in Metakaolin-Based Alkali-Activated Binders

Victorien Bienvenu Abanda Well *, Mattia Giovini, Francesco Genua, Isabella Lancellotti and Cristina Leonelli *

Department of Engineering "Enzo Ferrari", University of Modena and Reggio Emilia, 41121 Modena, Italy
* Correspondence: victorienbienvenu.abandawell@unimore.it (V.B.A.W.); cristina.leonelli@unimore.it (C.L.)

Supplementary Materials

The chemical analyses obtained via XRF spectrometry are reported in Table S1 in terms of main components (wt%) and trace elements (ppm, parts per million).

Table S1. Complete chemical composition of the five wastes as obtained from XRF results expressed in oxides weight % or in parts per million (ppm). The Loss on Ignition (LOI) was determined as mass loss after leaving the powder in oven 2 h at 1100°C.

Float glass polishing sludge (MO)			
Analyte	Compound formula	Concentration	Unit
	L.O.I.	15.20	%
Ti	Ti	105	ppm
Na	Na2O	7.93	%
Mg	MgO	3.84	%
Al	Al2O3	1.37	%
Si	SiO2	63.46	%
P	P	43	ppm
S	S	514	ppm
Cl	Cl	210	ppm
Ca	CaO	7.88	%
Ce	Ce	400	ppm
Mn	Mn	46	ppm
Fe	Fe	557	ppm
Cu	Cu	81	ppm
Zn	Zn	77	ppm
Sr	Sr	26	ppm
Zr	Zr	39	ppm
La	La	349	ppm
K	K	783	ppm
Mn,La,K,Zn,Cu,Fe,Ce,Cl,S,P,Ti		not detected	

Fine glass dust (PA)			
**Analyte**	**Compound formula**	**Concentration**	**Unit**
<H>	L.O.I.	8.35	%
Na	Na2O	10.36	%
Mg	MgO	2.98	%
Al	Al2O3	2.86	%
Si	SiO2	61.47	%
P	P2O5	0.17	%
S	SO3	0.48	%
Cl	Cl	0.30	%
K	K2O	0.69	%
Ca	CaO	10.55	%
Ti	TiO2	0.15	%
Cr	Cr	361	ppm
Mn	Mn	192	ppm
Fe	Fe2O3	1.37	%
Cu	Cu	146	ppm
Zn	Zn	592	ppm
Sr	Sr	113	ppm
Zr	Zr	113	ppm
Sn	Sn	350	ppm
Ba	Ba	381	ppm
Pb	Pb	193	ppm
Bi	Bi	125	ppm

Fly ash (black) (FAN)			
**Analyte**	**Compound formula**	**Concentration**	**Unit**
<H>	L.O.I.	2.74	%
Na	Na2O	1.65	%
Mg	MgO	3.22	%
Al	Al2O3	10.13	%
Si	SiO2	46.98	%
P	P2O5	1.98	%
S	SO3	1.45	%
Cl	Cl	870	ppm
K	K2O	8.12	%
Ca	CaO	18.47	%
Ti	TiO2	0.63	%
Cr	Cr	239	ppm
Mn	MnO	0.14	%
Fe	Fe2O3	4.11	%
Ni	Ni	153	ppm
Cu	Cu	143	ppm
Zn	Zn	347	ppm
Rb	Rb	154	ppm
Sr	Sr	667	ppm
Y	Y	33	ppm
Zr	Zr	289	ppm
Ba	Ba	444	ppm
Ce	Ce	365	ppm
Pb	Pb	81	ppm

Bottom ash (BA)			
**Analyte**	**Compound formula**	**Concentration**	**Unit**
<H>	L.O.I.	10.93	%
Na	Na2O	2.31	%
Mg	MgO	2.40	%
Al	Al2O3	8.33	%
Si	SiO2	23.07	%
P	P2O5	2.04	%
S	SO3	2.62	%
Cl	Cl	1.33	%
K	K2O	1.05	%
Ca	CaO	28.36	%
Ti	TiO2	1.14	%
Cr	Cr2O3	0.34	%
Mn	MnO	0.17	%
Fe	Fe2O3	14.74	%
Co	Co	175	ppm
Ni	Ni	242	ppm
Cu	CuO	0.28	%
Zn	ZnO	0.50	%
As	As	462	ppm
Br	Br	53	ppm
Rb	Rb	44	ppm
Sr	Sr	454	ppm
Zr	Zr	274	ppm
Sn	Sn	153	ppm
Ba	Ba	889	ppm
Ce	Ce	415	ppm
Pb	Pb	794	ppm

Fly ash (white) (FAB)			
**Analyte**	**Compound formula**	**Concentration**	**Unit**
<H>	L.O.I.	18.33	%
F	F	0.19	%
Na	Na2O	1.82	%
Mg	MgO	4.76	%
Al	Al2O3	2.39	%
Si	SiO2	6.19	%
P	P2O5	3.32	%
S	SO3	12.01	%
Cl	Cl	2.87	%
K	K2O	11.48	%
Ca	CaO	34.06	%
Ti	TiO2	0.13	%
Cr	Cr	109	ppm
Mn	MnO	0.20	%
Fe	Fe2O3	1.66	%
Ni	Ni	71	ppm
Cu	Cu	160	ppm
Zn	ZnO	0.33	%
As	As	53	ppm
Br	Br	167	ppm
Rb	Rb	218	ppm
Sr	SrO	0.13	%
Ba	Ba	345	ppm
Pb	Pb	269	ppm

Table S2: Compressive strength results completed with density and comments on defects. Tests were done on samples cured for 51 days.

Name	Strength (MPa)	Apparent Density (g/cm³)	Defects
GP0	18.41	1.40	none
S_PA5	20.07	1.38	none
S_PA10	15.3	1.42	none
S_PA25	11.46	1.32	none
S_PA50	10.08	1.26	Limited efflorescence
S-PA100	-	-	Deformation
S_BA5	15.66	1.24	Slight porosity increase
S_BA10	8.79	1.30	Slight porosity increase
S_BA25	-	-	Swelling
S_BA50	-	-	Swelling
S_BA100	-	-	Efflorescence and swelling
S_FAN5	16.37	1.36	none
S_FAN10	13.40	1.37	none
S_FAN25	19.23	1.40	none
S_FAN50	30.38	1.48	none
S_FAN100	-	-	cracking
S_FAB5	17.75	1.38	none
S_FAB10	16.58	1.43	none
S_FAB25	22.12	1.40	none
S_FAB50	21.03	1.49	none
S_MO5	16.51	1.34	none
S_MO10	17.90	1.40	none
S_MO25	22.34	1.40	none
S_MO50	14.82	1.51	none
S_MO100	-	-	Cracks and efflorescence
R3_PA5	15.44	1.41	none
R3_PA10	18.97	1.42	none
R3_PA15	18.24		
R3_FAB5	19.37	1.04	none
R3_FAB10	17.47	1.34	none
R3_FAB15	14.74	1.43	none
R3_MO5	20.34	1.43	none
R3_MO10	25.97	1.24	none
R3_BA5	14.32		
R3_BA10	22.84	1.37	none
R3_BA15	19.45	1.25	none
R3_FAN5	20.51	1.30	none
R3_FAN10	16.30	1.36	none
R3_FAN15	19.65	1.48	none

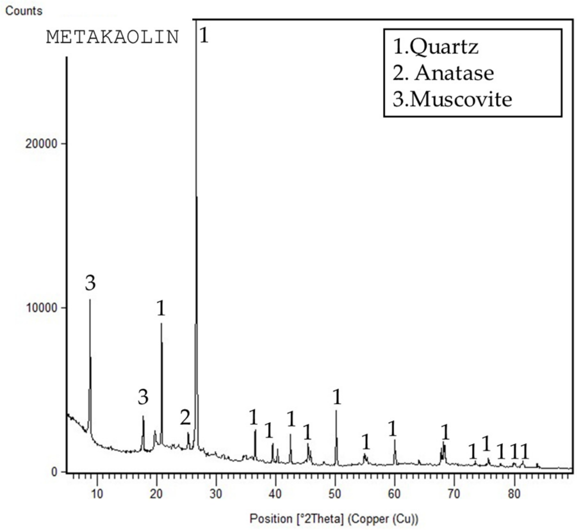

Figure S1: XRD pattern of pure metakaolin ARGICAL 1000.

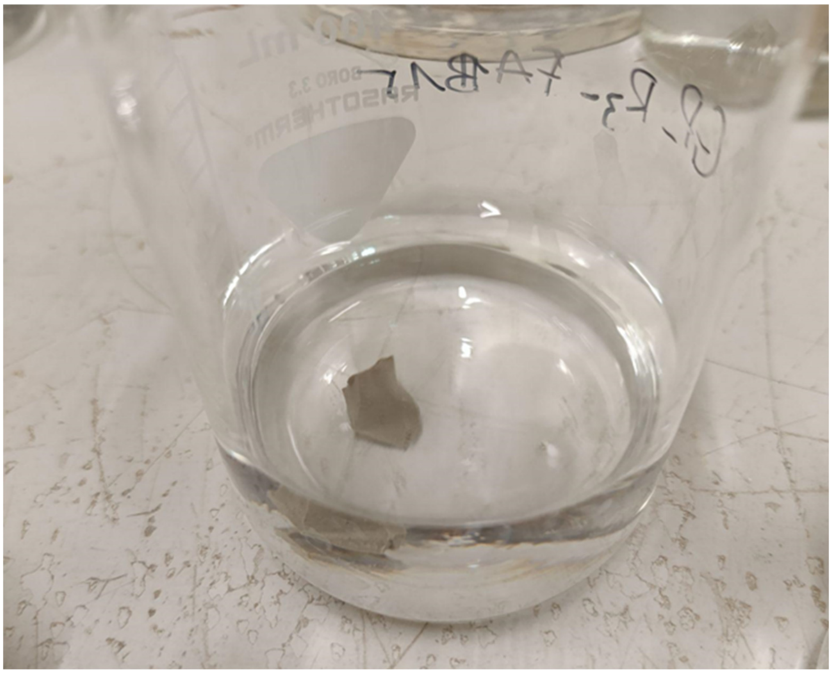

Figure S2: Example of alkali activated binder (R3-FAB15) after the integrity test: clear water and mechanical resistance of the solid indicate a good chemical stability of the geopolymers solid.

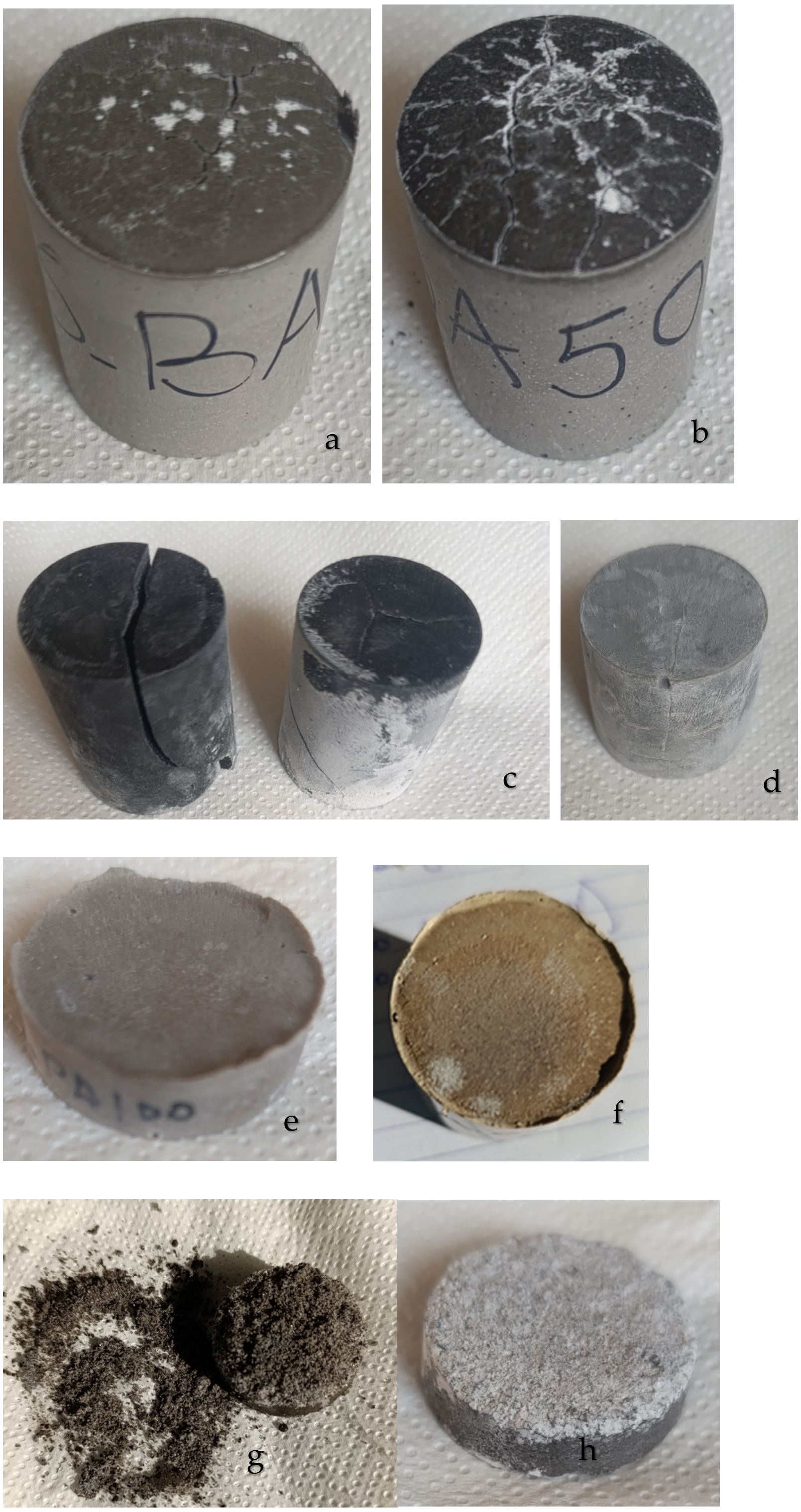

Figure S3: Appearance of the formulation which were discarded due to defects: a) Swelling observed for S_BA25, b) Swelling observed for S_BA50, c) large cracks observed and total rupture of the geopolymer for S_FAN100, d) cracks and efflorescence for S_MO100, e) deformation for S_PA100, f) efflorescence for S_PA50, g) and h) efflorescence for S_BA100.

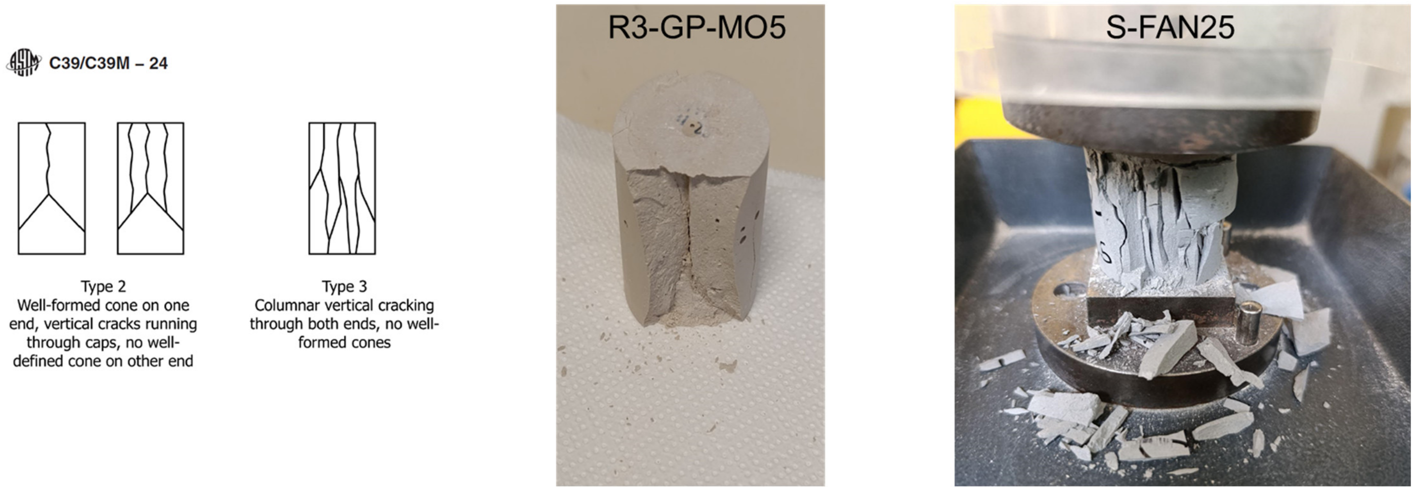

Figure S4: Fracture geometries compared to fracture pattern/type reported in ASTM C39.

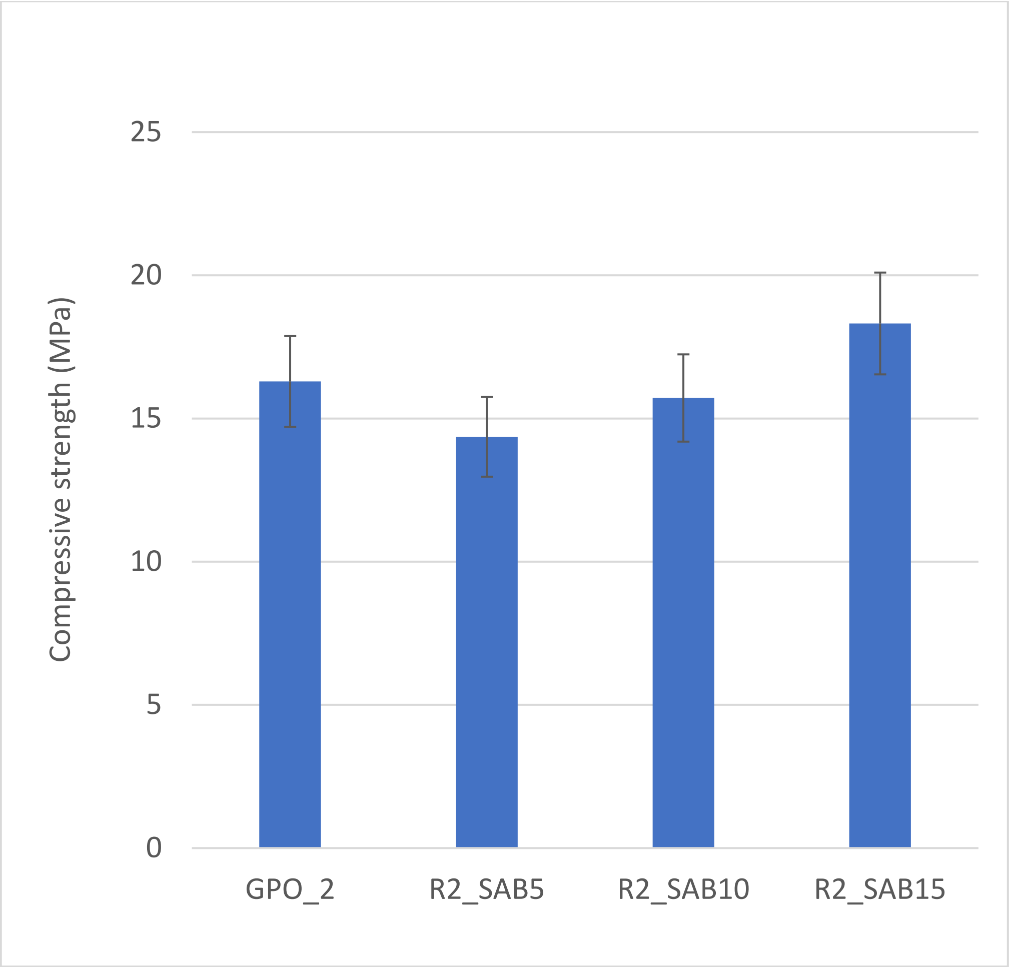

Figure S5: Effect of addition of standard sand on the mechanical strength of the reference GP0 formulation. The tests were performed after 28 days of curing.

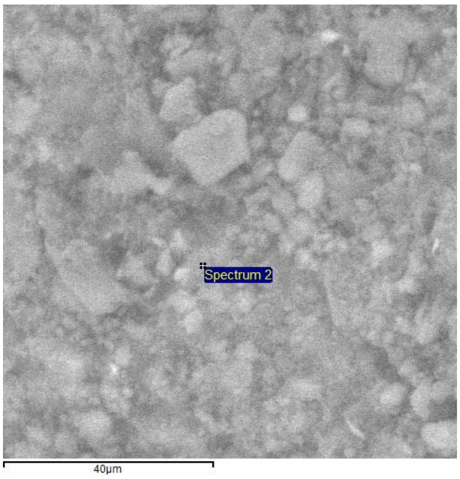

Element	Weight%	Atomic%	Compd%	Formula
C K	4.70	7.73	17.23	CO2
Na K	8.80	7.55	11.86	Na2O
Mg K	0.17	0.14	0.28	MgO
Al K	11.15	8.15	21.06	Al2O3
Si K	19.31	13.57	41.30	SiO2
K K	0.55	0.28	0.67	K2O
Ca K	0.54	0.27	0.76	CaO
Ti K	0.87	0.36	1.45	TiO2
Fe K	2.07	0.73	2.67	FeO
Au M	2.42	0.24	2.72	Au2O3
O	49.41	60.97		
Totals	100.00			

Figure S6: SEM image and chemical composition of the gel matrix obtained by EDS spectrum elaboration for sample R3-BA10.

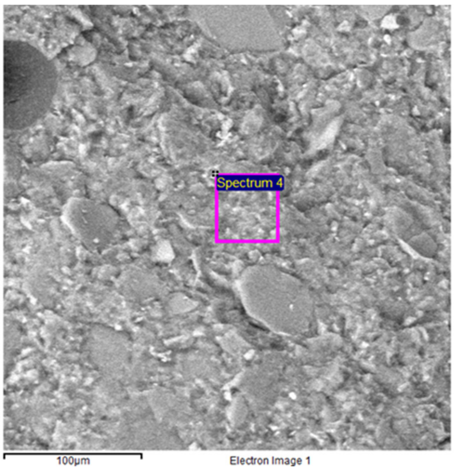

Element	Weight%	Atomic%	Compd%	Formula
C K	3.45	5.79	12.65	CO2
Na K	7.19	6.30	9.69	Na2O
Mg K	0.23	0.19	0.38	MgO
Al K	12.23	9.13	23.10	Al2O3
Si K	21.45	15.39	45.90	SiO2
K K	1.33	0.68	1.60	K2O
Ca K	1.08	0.54	1.50	CaO
Ti K	0.18	0.07	0.29	TiO2
Fe K	0.83	0.30	1.07	FeO
Au M	3.41	0.35	3.82	Au2O3
O	48.64	61.25		
Totals	100.00			

Figure S7: SEM image and chemical composition of the gel matrix obtained by EDS spectrum elaboration for sample R3-FAN15.

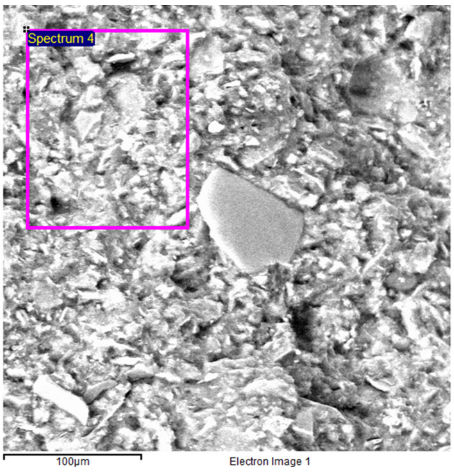

Element	Weight%	Atomic%	Compd%	Formula
C K	4.65	7.69	17.04	CO2
Na K	7.65	6.61	10.31	Na2O
Mg K	0.24	0.20	0.40	MgO
Al K	8.14	5.99	15.39	Al2O3
Si K	21.89	15.48	46.82	SiO2
K K	1.64	0.83	1.98	K2O
Ca K	2.34	1.16	3.27	CaO
Ti K	0.18	0.08	0.30	TiO2
Fe K	0.98	0.35	1.26	FeO
Au M	2.89	0.29	3.24	Au2O3
O	49.41	61.33		
Totals	100.00			

Figure S8: SEM image and chemical composition of the gel matrix obtained by EDS spectrum elaboration for sample S-FAN50.

% addition	SAND	MO	PA	FAN	BA	FAB
**0**	0	0	0	0	0	0
**5**	-11.9	19.6	-13.0	21.3	-15.3	14.5
**10**	-3.5	53.1	11.7	-2.7	35.3	3.8
**15**	12.4	n.d.	7.7	16.6	14.9	-12.1

Legend

- = Negative value
- = Zero
- = Moderate positive value
- = High positive value
- = Maximum positive value

Figure S9: Heatmap with numerical annotations illustrating the percentage increase or decrease in compressive strength of alkali-activated binders containing waste additions relative to the reference formulation GP0 with sand addition, enabling both trend identification and rapid comparison across the entire dataset.
